# Copy number variation of ribosomal DNA and *Pokey *transposons in natural populations of *Daphnia*

**DOI:** 10.1186/1759-8753-3-4

**Published:** 2012-03-05

**Authors:** Shannon HC Eagle, Teresa J Crease

**Affiliations:** 1 Department of Integrative Biology, University of Guelph, Guelph, ON N1G 2W1, Canada

**Keywords:** *Daphnia*, transposons, *Pokey*, ribosomal RNA genes, copy number variation, quantitative PCR

## Abstract

**Background:**

Despite their ubiquity and high diversity in eukaryotic genomes, DNA transposons are rarely encountered in ribosomal DNA (rDNA). In contrast, R-elements, a diverse group of non-LTR retrotransposons, specifically target rDNA. *Pokey *is a DNA transposon that targets a specific rDNA site, but also occurs in many other genomic locations, unlike R-elements. However, unlike most DNA transposons, *Pokey *has been a stable component of *Daphnia *genomes for over 100 million years. Here we use qPCR to estimate the number of 18S and 28S ribosomal RNA genes and *Pokey *elements in rDNA (r*Pokey*), as well as other genomic locations (g*Pokey*) in two species of *Daphnia*. Our goals are to estimate the correlation between (1) the number of 18S and 28S rRNA genes, (2) the number of 28S genes and r*Pokey*, and (3) the number of r*Pokey *and g*Pokey*. In addition, we ask whether *Pokey *number and distribution in both genomic compartments are affected by differences in life history between *D. pulex *and *D. pulicaria*.

**Results:**

We found differences in 18S and 28S gene number within isolates that are too large to be explained by experimental variation. In general, *Pokey *number within isolates is modest (< 20), and most are g*Pokey*. There is no correlation between the number of rRNA genes and r*Pokey*, or between r*Pokey *and g*Pokey*. However, we identified three isolates with unusually high numbers of both r*Pokey *and g*Pokey*, which we infer is a consequence of recent transposition. We also detected other rDNA insertions (r*Inserts*) that could be degraded *Pokey *elements, R- elements or the divergent *Pokey*B lineage recently detected in the *Daphnia *genome sequence. Unlike r*Pokey*, r*Inserts *are positively correlated with rRNA genes, suggesting that they are amplified by the same mechanisms that amplify rDNA units even though r*Pokey *is not. Overall, *Pokey *frequency and distribution are similar in *D. pulex *and *D. pulicaria *suggesting that differences in life history have no impact on *Pokey*.

**Conclusions:**

The possibility that many rDNA units do not contain a copy of both 18S and 28S genes suggests that rDNA is much more complicated than once thought, and warrants further study. In addition, the lack of correlation between r*Pokey*, g*Pokey *and rDNA unit numbers suggests that *Pokey *transposition rate is generally very low, and that recombination, in combination with natural selection, eliminates r*Pokey *much faster than g*Pokey*. Our results suggest that further research to determine the mechanisms by which *Pokey *has escaped complete inactivation by its host (the usual fate of DNA transposons), would provide important insights into transposon biology.

## Background

Transposable elements (TEs) are segments of DNA that can move or transpose around the genome [1]. Despite the fact that some have been co-opted for cellular functions by their host [2], TEs are generally considered to be detrimental because they can disrupt function when they insert into or near genes, or promote ectopic recombination, which can lead to chromosome rearrangements [3]. Moreover, their transposition may have energy costs [3] and the epigenetic mechanisms used by the host to control their expression can also alter gene function [4]. Even so, transposons are ubiquitous in eukaryotes and have become basic genomic components like exons, introns, telomeres and centromeres [5,6].

**Figure 1:**
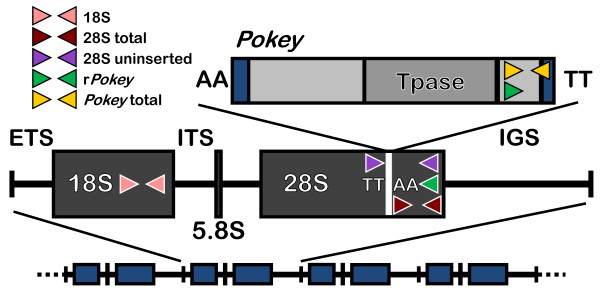
**Eukaryotic rDNA structure and location of qPCR primers to estimate Daphnia rDNA and Pokey number**. Primers are indicated by short arrows. *Pokey *inserts into a specific TTAA site in the 28S gene, which is duplicated at either end of the inserted element. 18S, 18S rRNA coding region; 28S, 28S rRNA coding region; 5.8S, 5.8S rRNA coding region; IGS, intergenic spacer; ETS, external transcribed spacer; ITS, internal transcribed spacer; Tpase, transposase gene. The darker vertical bars at either end of *Pokey *represent the Terminal Inverted Repeats.

Ribosomal (r)DNA is a multigene family composed of repeated units each containing an 18S, 5.8S and 28S rRNA gene, and spacers (Figure 1). Due to the highly repetitive nature of rDNA, it usually evolves in a concerted fashion such that rDNA units are more similar within than between species [7]. The primary mechanisms responsible for this phenomenon are thought to be unequal crossing over and gene conversion [7]. Given the constant turnover of rDNA units caused by this recombination, and the strong purifying selection that operates on the rRNA genes, it is somewhat surprising that some TEs insert specifically into rDNA [7]. On the other hand, it has been argued that its repetitive nature makes rDNA an ideal niche for TEs. For example, recombination can increase the number of rDNA units and thus provide new insertion sites, or it can remove inactive TEs to reopen sites into which active elements can insert. Moreover, rRNA genes are highly transcribed providing many opportunities for expression of TE-encoded genes [8]. TEs that specifically target rDNA typically insert into either the 18S or the 28S genes, which make the genes non-functional [9,10]. However, the number of rDNA units usually exceeds the minimum required for host viability, and thus insertion of TEs into some rDNA units may have little impact on host fitness [8,11]. Indeed, some species are able to recognize and preferentially deactivate rDNA units containing insertions [12]. The most extensively studied rDNA-specific TEs are the Class I non-LTR retrotransposons, R1 and R2 [11], which are common in arthropods. R2 and other related lineages have also been found in diverse animal phyla, including Chordata, Echinodermata, Plathyhelminthes, Rotifera and Cnidaria [9,10,13]. These TEs typically undergo long periods of stable, vertical inheritance and diverge congruently with their hosts [11].

*Pokey *is a Class II DNA transposon in the *piggyBac *superfamily that ranges in size from 4 to 10 kb and contains the terminal inverted repeats (TIRs) characteristic of this element class [14,15]. Many copies of *Pokey *encode a transposase whose ability to excise the element has been confirmed in a yeast excision assay [15]. *Pokey *was first identified in the rDNA of the cladoceran crustacean, *Daphnia pulex *[16] and is widespread in the subgenus *Daphnia *[17,18]. The only other taxa in which *Pokey *elements have been discovered are the silk moth, *Bombyx mori *[19] and the tunicate, *Ciona savignyi *[20]. *Pokey *is unique among DNA transposons in that it targets a specific TTAA site in the 28S genes of *Daphnia *(Figure 1) where it has undergone stable, vertical inheritance for millions of years [17]. Moreover, this TTAA site is only 4 nucleotides (nt) from the insertion site of R2, and 64 nt from the insertion site of R1. However, unlike R-elements, which tend to specialize on rDNA (but see [9]), *Pokey *also inserts into many other locations throughout the *Daphnia *nuclear genome [21-23], although only a single copy has been reported from the nuclear genomes of *B. mori *and *C. savignyi*.

*Daphnia *are freshwater crustaceans (Cladocera, Crustacea) that inhabit standing water from shallow, ephemeral puddles to deep stratified lakes. They typically reproduce by cyclic parthenogenesis in which production of direct-developing embryos by apomictic parthenogenesis alternates with the meiotic production of ephippial (diapausing) eggs that require fertilization. Males are produced apomictically and sex-determination is environmental. Populations that inhabit temporary bodies of water that either dry up or freeze during the year are re-established from ephippial offspring annually. In contrast, adults can persist year-round in permanent ponds and lakes, and recruitment of individuals from ephippial eggs may be sporadic. In addition, some lineages have completely lost the capacity for sexual reproduction and produce their ephippial eggs apomictically (obligate parthenogenesis).

Species in the *Daphnia pulex *complex typically inhabit ponds and small unstratified lakes that lack fish. However, one species in this complex, *Daphnia pulicaria*, has invaded large stratified lakes in North America [24], and is able to tolerate predation by fish by taking refuge in the cold hypolimnion during the day [25]. The transition to lake habitats has led to substantial changes in physiology and life history [26,27]. Even so, *D. pulicaria *produces viable hybrids with other members of the *D. pulex *species complex. Hybrids typically occur in ponds or disturbed, intermediate habitats, and reproduce by obligate parthenogenesis [28]. Despite this hybridization, lake populations of *D. pulicaria *remain genetically [29] and ecologically [26,27] distinct from the other species in the complex.

The occurrence of *Pokey *in both rDNA (r*Pokey*) and other genomic locations (g*Pokey*) in *Daphnia *provides a unique opportunity to study its distribution in different genomic compartments and under different modes of reproduction (with and without meiosis). For example, using the PCR-based technique TE Display [30], Valizadeh and Crease [21] and Schaack *et al. *[23] compared the relative load of g*Pokey *in cyclically and obligately parthenogenetic populations of *D. pulex *in North America. The results of both studies show that cyclical individuals carry, on average, more elements than obligate individuals, which is consistent with predictions about the effect of breeding system on TE dynamics [31]. However, it is not possible to estimate r*Pokey *or rRNA gene number with TE Display, so the dynamics of r*Pokey *have not yet been determined. On the other hand, Averbeck and Eickbush [32] measured R1 and R2 number in the rDNA of replicate laboratory lines of *Drosophila melanogaster *and found that R2 number remained relatively constant through time, but changes in R1 number were strongly correlated with changes in the overall size of the rDNA locus, which varied from 140 to 310 units after 400 generations of laboratory culture. Moreover, transposition of full-length elements accounted for most of the change in R1 number. This level of rRNA gene variation was also observed in replicate, clonally-propagated lines of *Daphnia obtusa*, in which the haploid number of 18S genes varied from 53 to 233 after only 90 generations [33].

**Figure 2:**
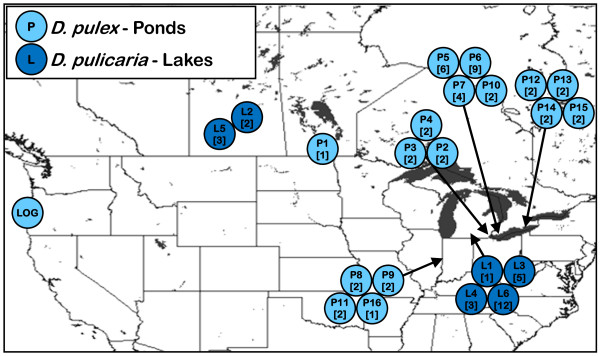
**Location of *D. pulex *and *D. pulicaria *populations sampled for this study**. *Daphnia pulex *were sampled from ponds (P) and *D. pulicaria *from lakes (L). Each circle contains the population code and the number of isolates analyzed in brackets.

In this study, we use quantitative PCR (qPCR) to measure the number of 18S and 28S rRNA genes, as well as r*Pokey *and g*Pokey *in 16 pond populations (43 isolates) of *D. pulex *and 6 lake populations (26 isolates) of *D. pulicaria *(Figure 2, Additional file 1). Our objectives are to estimate the correlation between (1) the number of 18S and 28S genes, (2) the number of 28S genes and r*Pokey*, and (3) the number of r*Pokey *and g*Pokey*. In addition, we ask if life history differences between *D. pulex *and *D. pulicaria *impact *Pokey *frequency and distribution. We expect a positive correlation between 18S and 28S gene number if most rDNA units are complete, although this has rarely been tested. Moreover, if rates of transposition and/or elimination by the host vary in response to the number of available insertion sites, we expect to observe a positive association between r*Pokey *and 28S gene number. We also expect a positive correlation between r*Pokey *and g*Pokey *number if rates of transposition and/or rates of element elimination are similar both inside and outside of rDNA. Finally, because rates of recruitment of sexually-produced offspring differ between pond populations, which are re-established each year from ephippial hatchlings, and lake populations, which often undergo extended periods of clonal reproduction, there may be more opportunities for transposition in pond populations and thus higher *Pokey *load.

## Results

We calculated the haploid number of rRNA genes (18S and 28S) and *Pokey *relative to two single-copy reference genes, *Tif *(a transcription initiation factor) and *Gtp *(a member of the RAB subfamily of small GTPases) [33]. The number of 28S was estimated in two regions: downstream of the *Pokey *insertion site (total 28S or t28S) and across the *Pokey *insertion site (uninserted 28S or u28S, Figure 1), which allowed us to determine if there are insertions present in that position that would not amplify with our *Pokey *primers (for example, R2). In addition, we estimated the number of r*Pokey *and total *Pokey *elements in the genome (Figure 1, Additional file 2).

We expect the number of one reference gene relative to the other to be close to 1, and this is often the case (Table 1, Additional file 2). However, values of *Tif *relative to *Gtp *(TG ratio) vary from 0.65 to 1.32 across the 69 *Daphnia *isolates, with a mean of 0.90 for *D. pulex *and 0.92 for *D. pulicaria *(Table 1). One explanation for the extreme values is that there are three copies of one gene instead of the two expected, in which case we expect a ratio of 0.67 (3 *Gtp*) or 1.5 (3 *Tif*). Further analysis (Additional file 3) suggests that there are three copies of *Gtp *in one isolate (TG ratio = 0.65) and three copies of *Tif *in the four isolates (TG ratios ≥ 1.25). Estimates of gene number based on the duplicated gene were adjusted by multiplying them by 1.5 in these isolates.

**Table 1:** *Pokey *and rRNA gene number in *Daphnia *from North America

			Number of genes mean/range							
			
Species	Number of populations	Number of isolates	*Tif:Gtp*^1^	18S	t28S^2^	r*Pokey*^3^	g*Pokey*^4^	r*Inserts*^5^	% *Pokey *in 28S^6^	% r*Inserts*^7^
*D. pulex*	16	43	0.90 0.65 to 1.31	221.0 94 to 489.5	260.0 88 to 724.5	2.1 0 to 12	9.6 4 to 18	21.1 0 to 113.5	17.2 0 to 63.2	6.5 0 to 28.6
*D. pulicaria*	6	26	0.92 0.70 to 1.32	215.1 97 to 444	266.1 109 to 654.5	6.6 0 to 44.5	9.5 4.5 to 24	14.9 0 to 76	27.1 0 to 79.2	4.6 0 to 19.7
*D. pulicaria *- 3^8^	6	23	0.93 0.70 to 1.32	217.3 97 to 444	273.9 109 to 654.5	2.7 0 to 7.5	8.9 4.5 to 18	16.9 0 to 76	21.2 0 to 57.7	5.2 0 to 19.7

1. Number of *Tif *genes relative to *Gtp *genes.

2. Denotes total 28S genes.

3. Denotes *Pokey *in 28S genes.

4. Denotes *Pokey *in other genome sites. Calculated as (total *Pokey *- r*Pokey*).

5. Denotes other rDNA inserts, calculated as (t28S-u28S-r*Pokey*) where u28S is uninserted 28S genes.

6. Calculated as (r*Pokey*/total *Pokey *x 100).

7. Calculated as (r*Inserts*/t28S × 100).

8. Results obtained after removing three *D. pulicaria *isolates with high *Pokey *load (see text)

### Variation in rRNA gene number

The haploid number of 18S varies from 94 to 489.5 across all 69 *Daphnia *isolates (Table 1), and the mean value is not significantly different between the two species (t = -0.26, df = 50, P = 0.79). An even wider range of variation was observed for 28S (88 to 724.5), but again, the mean value does not differ significantly between species (t = 0.18, df = 48, P = 0.86). Similarly, there is no significant difference between the mean number of 18S and 28S within each species (*D. pulicaria *t = -1.52, df = 43, P = 0.14; *D. pulex *t = -1.64, df = 74, P = 0.10).

**Figure 3:**
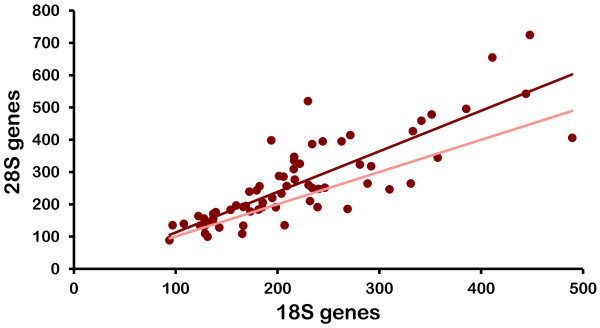
**Correlation between 18S and 28S rRNA genes in *D. pulex *and *D. pulicaria***. The haploid number of each gene is plotted. The lighter line was generated by plotting the number of 18S genes on both axes.

The estimates of 18S and 28S number within each isolate are significantly correlated (Table 2, Figure 3), but the slope of the line generated by plotting them relative to one another is 1.26, which is substantially higher than the expected value of 1. Moreover, 18S and 28S numbers are significantly different in 61% of the 69 isolates with 28S exceeding 18S in 76% of these cases (Additional files 2 and 4).

**Table 2:** Correlations between *Pokey *and rRNA gene number in *Daphnia *from North America

Species	X-axis	Y-axis	slope	y-intercept	R^2^	*P*-value	Figure
All isolates	18S	28S	1.257	-12.69	0.690	0.000	3

*D. pulex*	28S	u28S^1^	0.850	15.71	0.976	0.000	5a
	28S	r*Inserts*^2^	0.150	-17.84	0.556	0.000	5a
	28S	r*Pokey*^3^	-0.00002	2.14	0.000	0.995	5a
	g*Pokey*^4^	r*Pokey*	-0.016	2.29	0.0005	0.884	4b

*D. pulicaria*	28S	u28S	0.907	3.26	0.981	0.000	5b
	28S	r*Inserts*	0.104	-12.80	0.475	0.0001	5b
	28S	r*Pokey*	-0.011	9.54	0.020	0.493	5b
	g*Pokey*	r*Pokey*	1.475	-7.48	0.290	0.005	4b
*D. pulicaria-3*^5^	g*Pokey*	r*Pokey*	0.123	1.62	0.031	0.420	4b

1. Denotes uninserted 28S rRNA genes.

2. Denotes other rDNA inserts calculated as (t28S-u28S-r*Pokey*) where t28S is total 28S genes.

3. Denotes *Pokey *in 28S genes.

4. Denotes *Pokey *in other genome sites. Calculated as (total *Pokey *- r*Pokey*).

5. Results obtained after removing three *D. pulicaria *isolates with high *Pokey *load (see text).

### Variation in *Pokey *number

**Figure 4:**
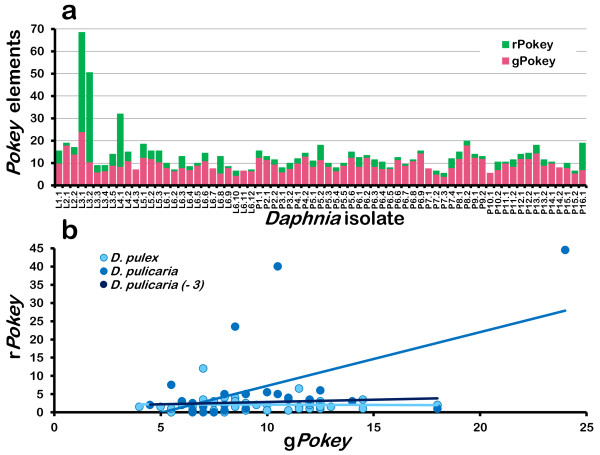
**Haploid *Pokey *number inside and outside of rDNA in *D. pulex *and *D. pulicaria***. L isolates are from lakes (*D. pulicaria*) and P isolates are from ponds (*D. pulex*). **(a) **g*Pokey *and r*Pokey *number in each isolate. r*Pokey *is inserted in 28S genes. g*Pokey *was calculated as (total *Pokey*- r*Pokey*). **(b) **Correlation between g*Pokey *and r*Pokey. D. pulicaria *(-3) refers to the analysis done after excluding the three isolates with high *Pokey *load (see text).

*Pokey *number in 28S (r*Pokey*) varies from 0 to 44.5, with a mean of 2.1 for *D. pulex *and 6.6 for *D. pulicaria *(Table 1, Figure 4a), but these differences are not significant (t = 1.96, df = 26, P = 0.06). The higher mean for *D. pulicaria *is due to three isolates with 23.5, 40 and 44.5 r*Pokey *(Figure 4a, Additional file 2). If we exclude these isolates, the mean decreases to 2.7 (Table 1). r*Pokey *number is 7.5 or fewer in all other *D. pulicaria *isolates, and three have none. Similarly, we did not detect r*Pokey *in three *D. pulex *isolates. Moreover, the haploid r*Pokey *number is only 0.5 in a total of eight isolates (Additional file 2), which means they have a single copy among all of their 28S genes. We tested these results in 26 isolates using end-point PCR, and they correspond with six exceptions in which we were not able to amplify r*Pokey *from isolates that have it based on qPCR. The qPCR estimate of r*Pokey *is 0.5 in three of these isolates, 1 in one isolate and 2 in two isolates.

*Pokey *number outside of rDNA (g*Pokey*) ranges from 4 to 24 with a mean of 9.6 in *D. pulex *and 9.5 in *D. pulicaria *(Table 1, Figure 4a), and this difference is not significant (t = -0.09, df = 40, P = 0.93). The same three *D. pulicaria *isolates with high r*Pokey *number also have high g*Pokey *number (8.5, 10.5, 24). If we exclude these isolates, mean g*Pokey *decreases to 8.9, which is lower than that in *D. pulex *(9.6) but not significantly different. Overall, g*Pokey *is present in all isolates, and, with the exception of the three unusual *D. pulicaria *isolates, it is much more numerous than r*Pokey *(Table 1, Figure 4a). On average, less than 28% of the *Pokey *elements in an isolate are located in 28S genes (Table 1).

There is no correlation between r*Pokey *and g*Pokey *number in *D. pulex*, but they are highly correlated in *D. pulicaria *(Table 2, Figure 4b). This correlation is mainly driven by the three unusual isolates with high *Pokey *load. We recalculated the correlation after excluding these isolates, and it is not significant.

### Variation in 28S gene insertion number

In general, the total number of 28S genes (t28S) exceeds the number of uninserted 28S genes (u28S), as expected. However, u28S is higher than t28S in 20 isolates. In addition, the sum of u28S plus r*Pokey *exceeds t28S in four isolates (Additional file 2). In these cases, we used the qPCR estimate of t28S and calculated u28S as (t28S-r*Pokey*).

**Figure 5:**
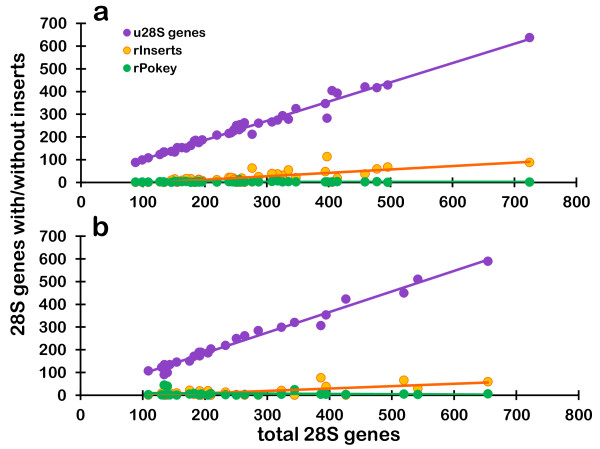
**Correlation between total 28S genes and genes with and without insertions**. **(a) ***Daphnia pulex*. **(b) ***Daphnia pulicaria*. u28S genes denotes uninserted 28S genes. r*Pokey *is inserted in 28S genes. The number of other rDNA insertions (r*Inserts*) was calculated as (t28S-u28S-r*Pokey*) where t is total 28S genes.

Differences in number between t28S and u28S (Table 1, Additional files 2 and 4) suggest that some genes contain insertions other than *Pokey*, or their *Pokey *elements do not bind to our qPCR primers. We refer to these as other rDNA inserts (r*Inserts*). r*Insert *number ranges from 0 to 113.5 in *D. pulex *(mean = 21.1) and from 0 to 76 in *D. pulicaria *(mean = 14.9, Table 1), and the means are not significantly different (t = -1.06, df = 60, P = 0.29). On average, r*Inserts *occur in less than 10% of 28S (Table 1). It is likely that some of the difference between t28S and u28S is due to experimental variation, and indeed this likely explains most cases where u28S exceeds t28S (as described above). However, experimental variation is not likely to be the only explanation because the slope of the line generated by plotting u28S relative to t28S is significantly lower than the expected value of 1 in both species, but the correlation between t28S and u28S is > 97% (Table 2, Figure 5).

There is a strong positive correlation (approximately 50%) between t28S and r*Inserts *in both species (Table 2), although r*Insert *number increases at a substantially slower rate (*D. pulex *slope = 0.15, *D. pulicaria *slope = 0.10, Figure 5) than does u28S. Moreover, these results are very different from the situation with r*Pokey*, which shows no correlation with t28S (Table 2, Figure 5) and is present in very low numbers in all but three *D. pulicaria *isolates (Figure 4).

### Comparison of rRNA gene and *Pokey *number based on qPCR and genome sequencing

Colbourne *et al. *[34] estimated the haploid number of rDNA units from the *D. pulex *genome sequence by comparing the depth of sequence coverage in the trace files across the entire rDNA repeat and the average single-copy gene. The estimate they reported is 435. In addition, Schaack *et al. *[22] searched the annotated genome sequence (which does not include rDNA) for *Pokey *using BLASTN and RepeatMasker. Their estimates based on these two approaches are 35 and 123, respectively.

In 2004, Schaack *et al. *[22] started a large number of clonally-propagated lines of the Log50 *D. pulex *isolate (whose genome was sequenced) from the lab culture that was established from the original female, collected in 2000. We obtained DNA extracted from two of these lines (Log50-11 and Log50-12) in 2006, approximately 40 generations after they were initiated, and estimated their *Pokey *and rRNA gene number using qPCR to compare them with estimates based on the genome sequence. The number of 18S is lower than 28S in both isolates (Table 3) with a ratio of 1:1.8 for Log50-11 and 1:1.5 for Log50-12. The mean number of 18S and 28S is 304 (Log50-12) and 421 (Log50-11), which is well within the range of variation observed within clonal *D. obtusa *lines over 90 generations [33]. Moreover, the Log50-11 estimate is very close to the value generated from the genome sequence (435).

**Table 3:** *Pokey *and rRNA gene number in two clonal lines of *Daphnia pulex*

	Gene Region						
	
Isolate^1^	18S	t28S^2^	u28S^3^	r*Pokey*^4^	g*Pokey*^5^	r*Inserts*^6 ^	*Tif:Gtp*^7^
Log50-11	301	540.5	445	2.5	37	93	0.84
Log50-12	245.5	362.5	349	4	25.5	9.5	0.88

1. Log50 is the isolate from which the *D. pulex *genome sequence was generated [34]. Log50-11 and Log50-12 are clonally-propagated lines derived from Log50 [22].

2. Denotes total 28S genes.

3. Denotes uninserted 28S genes.

4. Denotes *Pokey *in 28S genes.

5. Denotes *Pokey *in other genome sites. Calculated as (total *Pokey *- r*Pokey*).

6. Denotes other rDNA inserts, calculated as (t28S-u28S-r*Pokey*).

7. Number of *Tif *genes relative to *Gtp *genes

Our estimates of g*Pokey *number (37 and 25.5, Table 3) are also very similar to the estimate (35) obtained from the BLASTN search of the genome sequence. However, all three estimates are substantially lower than the one obtained from the genome sequence using RepeatMasker (123), suggesting that the similarity criteria used for the BLASTN search identified similar elements to the ones we detected with qPCR. Overall, our qPCR estimates are very similar to those obtained from the genome sequence.

## Discussion

### Variation in rRNA gene number

Variation in physiological responses to environmental heterogeneity has been linked with variation in rDNA copy number in diverse organisms (reviewed in [35]). Moreover, Paredes *et al. *[36] recently showed that differences in rDNA copy number have a significant effect on the level of expression of thousands of other genes in the *Drosophila *genome, which further reinforces the idea that rDNA copy number variation may be an important source of phenotypic and regulatory variation in natural populations. This is especially relevant for *Daphnia*, which is becoming an important model system for the study of genetic and physiological responses to environmental variation since publication of the genome sequence and development of other genomics tools [34].

In this study, we detected substantial variation in rRNA gene number within and between natural populations of *D. pulex *and *D. pulicaria*. The mean (approximately 220 for 18S, approximately 245 for 28S) and range is similar in both species with no values < 80 observed. However, the highest values are up to eight times larger than the smallest, which means that the diploid number of rRNA genes can vary by more than an order of magnitude among individuals of the same species. Such a large range of variation has been observed in natural populations of a diverse array of organisms, including plants, arthropods and vertebrates [35] and thus, *Daphnia *are not unusual in this regard.

Using computer simulations, Zhang *et al. *[8] showed that rDNA locus size is primarily a function of the rate of sister chromatid exchange, which creates high levels of variation among individuals and large rDNA loci. In contrast, high rates of interchromosomal exchange tend to reduce variation among individuals, and reduce the overall size of the rDNA locus. This model suggests that the highly variable number of rRNA genes in *D. pulex *and *D. pulicaria *results from higher rates of intrachromosomal than interchromosomal exchange, and this is indeed consistent with previous studies of rDNA variation in *Daphnia *[33,37,38]. Although rates of rDNA recombination have not been estimated in *D. pulex *or *D. pulicaria*, a rate of 0.02 to 0.06 events per generation was obtained in the congeneric species, *D. obtusa *[33], which is at the high end of rates estimated for rDNA in other organisms (10^-2 ^to 10^-5 ^[33]).

It is generally assumed that rDNA loci primarily contain canonical units consisting of one copy of each rRNA gene plus spacers (Figure 1), but this has rarely been tested. Based on this assumption, we expected our estimates of 18S and 28S number to be very similar within isolates, and they often are (Additional files 2 and 4). However, there are also a substantial number of isolates in which one gene (usually 28S) significantly outnumbers the other (Additional files 2 and 4) suggesting that experimental variation is not likely to account for all these differences. This conclusion is supported by the discovery of unusual rDNA configurations in humans. For example, Caburet *et al. *[39] used fluorescent *in situ *hybridization (FISH) and Southern blotting to show that up to one-third of the rDNA units in human fibroblast cell lines consist of head-to-head and tail-to-tail palindromic rearrangements of 18S and 28S. Moreover, these noncanonical units sometimes occur in clusters, which is consistent with their amplification by unequal sister chromatid exchange between rDNA units. In addition, Zafiropoulosa *et al. *[40] used qPCR to measure the number of 18S, 5.8S and 28S in adipose tissue samples from humans and found significant differences between genes within individuals. They concluded that these differences were a consequence of unequal recombination events initiating between the 18S and 5.8S and, subsequently, eliminating part of an rDNA unit. These studies suggest that our observation of large differences in 18S and 28S number in some *Daphnia *isolates may not be an artefact of qPCR analysis, and thus warrants further study.

### Variation in *Pokey *number

On average, we found only 2 to 3 r*Pokey *elements per haploid genome (maximum of 12 with three exceptions discussed below, Table 1). We also found isolates in which r*Pokey *is completely absent, which is also the case in previous studies [21,41]. In contrast, g*Pokey *is present in all isolates that have been analyzed in this and previous studies [21,23,41]. Indeed, over 75% of *Pokey *elements are located outside of 28S genes in most isolates. Thus, even if all the genes containing *Pokey *insertions were eliminated from rDNA, active g*Pokey *elements could eventually "recolonize" it. However, despite the higher number of g*Pokey*, they are also fairly constrained; there are usually less than 10 copies per haploid genome. These results are similar to those obtained by Valizadeh and Crease [21] using TE Display. They estimated *Pokey *number per diploid genome to be from 1 to 12 in 83 isolates of *D. pulex *from the Midwest US and Ontario. However, these are likely to be underestimates because Valizadeh and Crease [21] used an annealing temperature of 55°C. Vergilino [18] analyzed *Daphnia *isolates using TE Display, but used an annealing temperature of 50°C. We analyzed a few isolates from his study using an annealing temperature of 55°C, and obtained about half as many fragments as he did (data not shown). If the g*Pokey *estimates from Valizadeh and Crease [21] were doubled, they would be even closer to the ones we obtained with qPCR (Table 1).

The low number of both r*Pokey *and g*Pokey *in most isolates is consistent with the hypothesis that *Pokey *activity is generally low. Recombination among rDNA units further increases the rate at which r*Pokey *is eliminated, and can eventually eliminate it altogether in some isolates. However, if *Pokey *has been completely inactive for a substantial period of time in either species, we would not expect to find any r*Pokey*, although g*Pokey *could persist and even go to fixation by drift in some sites if it did not have deleterious effects on gene structure or function. Nevertheless, we identified three isolates with unusually high numbers of both r*Pokey *and g*Pokey*, and indeed, these isolates are responsible for the positive correlation between them in *D. pulicaria *(Figure 4b). It is unlikely that the high r*Pokey *number in these isolates is a consequence of large changes in the size of the rDNA locus as the two isolates with 40 or more r*Pokey *have less than 150 28S genes per haploid genome (Additional file 2). Moreover, such changes in rDNA would have little or no impact on g*Pokey *number. Thus, the most likely explanation for the high *Pokey *number in these isolates is a recent increase in transposition activity.

There are several mechanisms that could explain the putative increase in *Pokey *activity in these isolates. For example, it has been shown that some TEs are activated during a response to stress ([42] and references within). Alternatively, mutations could have occurred in *Pokey *that increase its ability to transpose or allow it to avoid silencing by the host, or in the host that decrease its ability to silence *Pokey *[3,43]. A similar scenario was suggested by Eickbush and colleagues [12,44], who showed that levels of R2 transcription can vary up to 100-fold in natural populations of *Drosophila*. They explained this by suggesting that R1 and R2 serve as foci for the formation of heterochromatin, which deactivates rDNA units. Because active rDNA units in *Drosophila *tend to occur in contiguous blocks [45,46], individuals are best able to silence these elements when they are clustered with one another in the rDNA array. Conversely, if they have been interspersed with uninserted, functional 28S genes by recombination, they are less likely to be silenced and thus show higher rates of transposition.

We do not know how *Pokey *elements are distributed in the rDNA of *Daphnia*, but Glass *et al. *[47] suggested that they are likely to be clustered based on patterns of sequence variation among 28S with and without *Pokey *insertions. In contrast, FISH analysis of one isolate with an IGS and a *Pokey *probe suggests that its *Pokey *elements are dispersed throughout the rDNA array [34]. Thus, it is not clear if the relationship between element activity and distribution suggested for R-elements in *Drosophila *will also be the case for r*Pokey *in *Daphnia*. However, this could be tested by analyzing the same individuals (or their clonally produced offspring) using both FISH and qPCR.

### Variation in 28S gene insertion number

Based on analysis of *D. pulex *rDNA cloned into a phage vector, Sullender [16] estimated that r*Pokey *elements occupy approximately 10% of the 28S genes. Based on our qPCR analyses, this is a substantial overestimate. However, even though r*Pokey *generally occupies about 1% of the 28S in an isolate, r*Inserts *occupy 5 to 7% of these genes, on average. Furthermore, unlike r*Pokey*, r*Insert *number is significantly correlated with t28S number (Table 2, Figure 5), suggesting that r*Inserts *are amplified by the same mechanism that amplifies u28S, and/or their transposition rate increases as u28S number increases. This same pattern was observed by Averbeck and Eickbush [32] for R1 in the replicate lines of *D. melanogaster*, although the slope of the line generated by plotting the total number of rRNA genes relative to the number of R1 insertions was 0.27, which is two to three times higher than our result of 0.10 for *D. pulicaria *and 0.15 for *D. pulex *(Table 2). Averbeck and Eickbush [32] argued that R2 insertions, whose number remained low (as does r*Pokey *in *Daphnia*), were excluded from recombination events among rDNA units, while R1 elements were underrepresented but still included, which allowed them to increase as the size of the rDNA locus increased (as do r*Inserts *in *Daphnia*). Even so, R1-inserted 28S did not increase at the same rate as u28S, suggesting that their amplification was somehow constrained. The simulation study of Zhang *et al. *[8] provides an explanation for this behavior. When simulations included elimination of chromosomes with low numbers of u28S by natural selection, recombination among rDNA units tended to increase u28S faster than inserted (i)28S.

Glass *et al. *[47] sequenced approximately 850 nt of 28S downstream of the *Pokey *insertion site in 20 isolates of *D. pulex *and found that variation was higher among genes with *Pokey *than those without. This is consistent with relaxed selection on these presumably non-functional 28S, which allows them to accumulate nt substitutions and short indels that would normally be deleterious. It is also consistent with the hypothesis that the presence of *Pokey *inhibits recombination between i28S and u28S. This inhibition may also explain why there is no correlation between r*Pokey *and t28S (Table 2, Figure 5), and why selection is not as efficient at removing 28S genes with *Pokey *insertions, which are much more deleterious than a single nt substitution or short indel.

It is possible that some of the r*Inserts *we detected in *Daphnia *28S are R1 and/or R2, but this seems unlikely. First, the insertion site for R1 is not located between the qPCR primers that span the *Pokey *TTAA insertion site. Second, we have not been able to amplify R2 (whose insertion site is 4 nt downstream of the TTAA) from some of the *D. pulex *and *D. pulicaria *isolates analyzed in this study using degenerate primers that have been used successfully in a wide range of arthropod species [48]. A more likely possibility is that the r*Inserts *we detected are degraded or divergent *Pokey *elements, and indeed, a second *Pokey *lineage has recently been identified in the *D. pulex *genome sequence [15]. Based on its similarity to a divergent lineage that was previously identified in *D. obtusa *[17], this lineage has been designated *Pokey*B. In addition, two types of miniature inverted-repeat transposable elements (MITEs) were also identified in the genome sequence. These MITEs are approximately 750 nt in length, and one of them (m*Pok*1) has TIRs similar to the original *Pokey *lineage, which we now designate as *Pokey*A, while the other (m*Pok*2) has TIRs similar to *Pokey*B [15].

Sequence alignment of the four groups of elements suggests that our qPCR primers will not amplify *Pokey*B or m*Pok*2, but they should bind to m*Pok*1. Thus, it is possible that some of the r*Inserts *are members of these two groups. This is supported by the fact that we have been able to amplify *Pokey*B and m*Pok*2 from the 28S of some of our *D. pulex *and *D. pulicaria *isolates using a forward primer that is specific to them and a reverse primer in the 28S (data not shown). So far, we have not been able to amplify m*Pok*1 from 28S, although it is usually present elsewhere in the genome.

Another possibility is that r*Inserts *are partial *Pokey *elements generated by recombination events that deleted part of the element and possibly part of the rDNA unit in which it resides. This would explain why numbers of *Pokey*A and 18S are similar for both Log50 isolates, but Log50-11 has approximately 240 more copies of 28S than 18S. These additional 28S could have inserts (Table 3), or they may be recombinants that did not amplify with the primers spanning the *Pokey *insertion site. The fact that *Pokey *contains sequences derived from the ribosomal IGS [14,15] supports the idea that aberrant rDNA units with (partial) *Pokey *insertions could have been created by recombination between r*Pokey *and rDNA. If this is the case, we expect these aberrant rDNA units to be deleterious and thus eliminated by natural selection before they expand to such high number. On the other hand, they could persist within populations for a considerable period of time if (1) they are clustered, which could occur if they were amplified by unequal sister chromatid exchange between rDNA units that are offset by one or a few copies, or (2) if they are not transcribed, either because their promoters were deleted by recombination (as suggested in [39] for the human fibroblasts), or they ended up in a region of the rDNA array that is particularly "attractive" to the heterochromatinization machinery (as suggested in [44] for R1 and R2).

Whatever the nature of r*Inserts *in *Daphnia *28S genes, it is clear that their dynamics are somewhat different from those of *Pokey*A. The latter behave like R2 in the replicate *D. melanogaster *lines [32], while the r*Inserts *behave more like R1. Averbeck and Eickbush [32] attributed the differences between these two elements to two factors; differences in rates of participation in recombination events and differences in transposition rate, which were estimated to be much higher for R1 than R2. If *Daphnia *r*Inserts *are indeed *Pokey*B or m*Pok*2, we would expect them to have a higher transposition rate than *Pokey*A. We are in the process of testing this prediction using yeast excision assays, which have shown that the *Pokey*A transposase is active [15]. If *Pokey*B does have a higher excision rate, we would also expect it to be more numerous than *Pokey*A in other genomic locations as well as rDNA. We also predict that m*Pok*2 will be more numerous in 28S than are full length *Pokey*A or *Pokey*B for two reasons. First, MITEs are often found to occur in higher number than the full-length elements whose transposase they use to move [49]. Second, the much shorter length of m*Pok*2 may reduce or eliminate the bias against recombination between i28S and u28S. This could contribute to the significant correlation between t28S and r*Inserts *(Table 2, Figure 5). In order to test these predictions, we are now in the process of developing qPCR primers to quantify each of the *Pokey *and MITE groups separately, both in and outside of 28S genes.

### Impact of life history variation on rDNA and *Pokey *dynamics

Previous studies [21,23,37,41,47] have shown that loss of sexual reproduction in obligately parthenogenetic lineages of *D. pulex *does impact both rDNA and g*Pokey *dynamics. In this study, we compared two *Daphnia *species whose life histories differ in several ways, including timing and frequency of sexual reproduction, brood size, juvenile growth rate and life span [26]. Overall, patterns of rDNA and *Pokey *number variation are virtually indistinguishable in the two species (Table 1, Figures 4 and 5). Thus, it seems that their life history differences have little, if any, impact on *Pokey *dynamics, most likely because "a little bit of sex is nearly as good as a lot" [50]. The only exception is the three D. *pulicaria *isolates with unusually high r*Pokey *and g*Pokey *loads, which we suggest is a consequence of recent transposition activity. Further analysis of isolates from these populations would likely provide additional insights into *Pokey *dynamics.

## Conclusions

It is clear that rDNA is very dynamic and much more complicated than once thought. In this study, we observed substantial variation in rDNA copy number among *Daphnia *isolates, which is consistent with previous studies suggesting that sister chromatid exchange is more frequent than interchromosomal exchange in *Daphnia *rDNA. Moreover, we sometimes found substantial differences in 18S and 28S number within isolates, which is not likely to be explained entirely by experimental variation and thus warrants further study. In general, there are less than 20 *Pokey *elements per haploid genome and most are g*Pokey*. This suggests that transposition rates are generally very low and that recombination, in combination with natural selection, eliminates r*Pokey *faster than g*Pokey*. Even so, three isolates of *D. pulicaria *have unusually high numbers of both r*Pokey *and g*Pokey*, which we suggest is due to a recent increase in transposition activity. We also detected other rDNA insertions (r*Inserts) *that could be degraded *Pokey *elements, R- elements or members of the divergent *Pokey*B lineage that was recently detected in the *D. pulex *genome sequence. Although r*Pokey *number is not correlated with t28S number, r*Insert *number is, suggesting that they are amplified by the same mechanisms as rDNA units while r*Pokey *is not. Overall, we observed no impact of life history differences between *D. pulex *and *D. pulicaria *on the dynamics of either r*Pokey *or g*Pokey*.

## Methods

### *Daphnia *samples and DNA extractions

A total of 69 *Daphnia *isolates collected from 16 ponds (*D. pulex*) and 6 lakes (*D. pulicaria*) were included in this study (Figure 2, Additional file 1). Ponds were sampled by skimming a dip net just under the surface of the water. Lakes were sampled by towing a net behind a small boat. Clonally-propagated lines were established from single females and grown in 200 mL of dechlorinated tap water at room temperature. Cultures were fed either live *Scenedesmus *or frozen *Nannochloropsis *algae (Landlocked Mariculture LLC, Toronto, Ontario, Canada). Genomic DNA was extracted from multiple individuals from each line using the phenol:chloroform method [51] or the AquaGenomics extraction kit with the manufacturer's tissue protocol (MultiTarget Pharmaceuticals LLC, Salt Lake City, Utah, USA). DNA concentrations were estimated using a NanoDrop^® ^ND-8000 spectrophotometer (Wilmington, Delaware, USA) and ranged from 1 to 1,500 ng/μL.

Lab isolates were identified as *D. pulex *or *D. pulicaria *by PCR-amplifying and sequencing an approximately 700 nt fragment of the mitochondrial *NADH dehydrogenase *5 (ND5) gene [52]. The breeding system (cyclic or obligate parthenogenesis) of most lab-reared isolates was determined by examination of diapausing egg cases (ephippia) produced in the absence of males. While cyclical parthenogens often release empty ephippial cases unless the eggs have been fertilized, obligate parthenogens deposit eggs into ephippia even in the absence of males [53].

### qPCR

We used the ΔC_T _quantitative (q)PCR method to estimate haploid copy number by comparing the rate of amplification of a multicopy gene to that of two single-copy genes as in McTaggart *et al. *[33]. The copy number of 18S, t28S, u28S, r*Pokey*, total *Pokey, Tif *and *Gtp *were estimated using seven pairs of primers (Figure 1, Additional file 5). We generated standard curves (Additional file 6) to validate each primer pair and determine its percent amplification efficiency (PAE).

qPCR reactions were 20 μL in volume, with 1X Power SYBR^® ^Green PCR Master Mix (Applied Biosystems, Foster City, California, USA), 0.25 pmol of each primer, and approximately 10 ng of template. Reactions were run on the StepOnePlus™ Real-Time PCR System (Applied Biosystems). The PCR program was as follows: 95°C for 10 minutes, 40 cycles of 95°C for 15 sec and 60°C for 1 minute. After the 40 cycles were complete, a dissociation curve was created during one additional cycle by increasing the temperature from 60°C to 95°C in increments of 0.3°C. All reactions were run in triplicate.

The baseline was set automatically by the StepOne v2.0 software (Applied Biosystems). The threshold was set based on amplicon size as larger amplicons bind more SYBR Green and thus produce more fluorescence. This has little effect when gene number is low, but the effect is substantial at high gene numbers. Thus, we set the threshold to 0.2 for the smallest amplicon of 50 bp, and calculated the threshold for larger amplicons as 0.2 × 2^(1-(50/length in bp)). If the standard deviation of the triplicate mean C_T _value was larger than 0.2, we excluded the most extreme replicate. However, if there was no clear outlier, we used all three C_T _values in the analysis.

We calculated gene number according to [33] with the PAE correction of [54] as 2^-ΔCT ^where ΔC_T _is ((C_T _× PAE _multicopy gene_)-(C_T _× PAE _single-copy gene_)). We used all combinations of C_T _values from multi-copy and single-copy gene triplicates to generate a total of 18 estimates ((3 × 3) + (3 × 3)) when all values were included (Additional file 7). These 18 estimates were averaged to give the mean and standard deviation of haploid copy number for each multi-copy gene in each isolate. The haploid numbers were rounded up or down to the nearest 0.5. We used Microsoft Excel (Richmond, Washington, USA) to do correlation and regression analyses, and t-tests. We used the sequential Bonferroni technique of Rice [55] to adjust the significance level (0.05) for t-tests comparing 18S and 28S number within isolates.

## Abbreviations

18S: 18S rRNA gene; 28S: 28S rRNA gene; bp: base pair; C_T_: threshold cycle; ETS: external transcribed spacer; FISH: fluorescent *in-situ *hybridization; g*Pokey: Pokey *elements found outside of rDNA; i28S-inserted 28S rRNA gene; IGS: intergenic spacer; ITS: internal transcribed spacer; kb: kilobase pair; LTR: long-terminal repeat; MITE: miniature inverted-repeat transposable element; nt: nucleotide; PAE: percent amplification efficiency; qPCR: quantitative PCR; rDNA: ribosomal DNA; r*Inserts*: rDNA insertions other than r*Pokey*; r*Pokey: Pokey *elements found in rDNA; rRNA: ribosomal RNA; t28S: total 28S rRNA genes; TE: transposable element; TG ratio: *Tif *to *Gtp *ratio; TIR: terminal inverted repeat; u28S: uninserted 28S rRNA gene.

## Competing interests

The authors declare that they have no competing interests.

## Authors' contributions

SHCE collected and analyzed the data. TJC conceived the study and supervised the work. Both authors wrote the manuscript and approved the final version.

## Supplementary Material

Additional file 1 **Population location and sample size**. This PDF file provides sample size, latitude and longitude for the 22 *Daphnia *populations sampled. Click here for file

Additional file 2 **Haploid rRNA gene and *Pokey *number**. This PDF file provides estimates (and standard deviation) of rRNA gene and *Pokey *number for all *Daphnia *isolates in this study. *Daphnia pulex *were collected from ponds (P) and *D. pulicaria *from lakes (L). Click here for file

Additional file 3 ***Tif *and *Gtp *cloning experiment**. This PDF file describes the cloning and sequencing of *Tif *and *Gtp *genes from four *Daphnia *isolates with a range of *Tif:Gtp *ratios. The purpose of this work was to determine if isolates with very low or very high *Tif:Gtp *ratios possess three alleles at one of the loci. Click here for file

Additional file 4 **Histograms of haploid rRNA gene and insertion number**. This is a PDF file. **(a) **Haploid 18S and 28S gene number in each *Daphnia *isolate. Vertical lines are standard errors. Differences that are not significant after sequential Bonferroni correction are indicated by "ns". **(b) **Haploid number of r*Pokey *and 28S with and without inserts in each *Daphnia *isolate. u28S are uninserted 28S genes, r*Pokey *are inserted in 28S, r*Inserts *are inserts other than r*Pokey *in 28S. The number of r*Inserts *was calculated as (total 28S-uninserted 28S-r*Pokey*). Click here for file

Additional file 5 **qPCR primers**. This PDF file provides sequences for qPCR primers, as well as the threshold value and the percent amplification efficiency (PAE) for each primer pair. Click here for file

Additional file 6 **Standard curve analysis for qPCR primers**. This PDF file provides details of the standard curve experiments used to validate the qPCR primers, including a description of the plasmid clones used as templates. Click here for file

Additional file 7 **C_T _values for all qPCR reactions**. This Excel spreadsheet provides C_T _values for all *Daphnia *isolates and qPCR amplicons. Replicates that were omitted from the analyses are highlighted in grey. A template for calculating gene number using the ΔC_T _method is also provided, with sample calculations for isolate P6.7. Click here for file
