# Supplementary material for: Copy number variation of ribosomal DNA and Pokey transposons in natural populations of Daphnia
Source: Mob DNA. 2012 Mar 5;3:4. doi: 10.1186/1759-8753-3-4 (PMC3315735; doi:10.1186/1759-8753-3-4)
Supplement: Additional file 1 — Population location and sample size. This PDF file provides sample size, latitude and longitude for the 22 Daphnia populations sampled. [file 1759-8753-3-4-S1.PDF]

Additional File 1. Daphnia populations sampled for this study						
**Species**	**Population**	**Population Code**	**Number of Individuals**	**Province/ State**	**Latitude**	**Longitude**
D. pulicaria	Three Lakes Two	L1	1	MI	42.21	-85.26
D. pulicaria	Humboldt Lake	L2	2	SK	52.13	-105.13
D. pulicaria	Lawrence Lake	L3	5	MI	42.26	-85.21
D. pulicaria	Mill Lake	L4	3	MI	42.27	-85.15
D. pulicaria	River Lake	L5	3	SK	50.28	-106.27
D. pulicaria	Warner Lake	L6	12	MI	42.28	-85.31
**D. pulicaria**	**Total**		**26**			
D. pulex	Prairie Pond 22	P1	1	MB	49.64	-98.24
D. pulex	Ann Arbor 71	P2	2	MI	42.19	-83.58
D. pulex	Ann Arbor 72	P3	2	MI	42.20	-83.72
D. pulex	Bethel Church 2	P4	2	MI	42.18	-83.90
D. pulex	Canard 2	P5	6	ON	42.13	-82.99
D. pulex	Canard 3	P6	9	ON	42.12	-82.98
D. pulex	Canard 3i	P7	4	ON	42.12	-82.98
D. pulex	Circle Pond	P8	2	IL	40.07	-87.92
D. pulex	Dead Cow Pond	P9	2	IL	40.07	-87.92
D. pulex	Disputed Road Pond	P10	2	ON	42.18	-83.04
D. pulex	Dog Shit Pond	P11	2	IL	40.07	-87.92
D. pulex	Long Point Pond 2	P12	2	ON	42.65	-80.49
D. pulex	Long Point Pond 5	P13	2	ON	42.67	-80.40
D. pulex	Long Point Pond 8B	P14	2	ON	42.68	-80.45
D. pulex	Long Point Pond 9A	P15	2	ON	42.67	-80.48
D. pulex	Portland Arch Pond 88	P16	1	IN	40.22	-87.33
**D. pulex**	**Total**		**43**			
