# Supplementary material for: Copy number variation of ribosomal DNA and Pokey transposons in natural populations of Daphnia
Source: Mob DNA. 2012 Mar 5;3:4. doi: 10.1186/1759-8753-3-4 (PMC3315735; doi:10.1186/1759-8753-3-4)
Supplement: Additional file 2 — Haploid rRNA gene and Pokey number. This PDF file provides estimates (and standard deviation) of rRNA gene and Pokey number for all Daphnia isolates in this study. Daphnia pulex were collected from ponds (P) and D. pulicaria from lakes (L). [file 1759-8753-3-4-S2.PDF]

Additional File 2. *Pokey* and rRNA gene number in each *Daphnia* isolate.

Isolate Code	Calculate Directly from Ct values													Estimated by subtraction			Percent			p-value differences 18S vs 28S [2]
	**18S**	**18S SD**[1]	**Total 28S**	**Total 28S SD**	**Uninserted 28S** (unadjusted)	**Uninserted 28S** (adjusted)	**Uninserted 28S SD**	**r*Pokey***	**r*Pokey* SD**	**Total *Pokey***	**Total *Pokey* SD**	**Tif to Gtp**	**Tif to Gtp SD**	**Inserted 28S** (total 28S - uninserted 28S)	**r*Inserts*** (total 28S - uninserted 28S - rPok)	**g*Pokey*** (total Pok - rPok)	**% rPok** (rPok/total Pok *100)	**% inserted 28S** (inserted 28S/total 28S *100)	**% r*Inserts*** (other inserted 28S/total 28S *100)	
L1.1	411.0	90.6	654.5	128.9	590.0	590.0	115.6	5.5	1.1	15.5	3.1	0.70	0.06	64.5	59.0	10.0	35.5	9.9	9.0	2.70E-07
L2.1	206.0	34.8	285.5	37.0	295.5	284.5	46.7	1.0	0.2	19.0	2.5	0.82	0.10	1.0	0.0	18.0	5.3	0.4	0.0	1.24E-07
L2.2	333.0	29.1	426.5	38.1	458.0	423.5	33.5	3.0	0.3	17.0	1.4	1.02	0.08	3.0	0.0	14.0	17.6	0.7	0.0	4.76E-08
L3.1	129.0	19.2	134.5	20.6	103.0	90.0	14.6	44.5	7.5	68.5	9.6	0.79	0.08	44.5	0.0	24.0	65.0	33.1	0.0	**0.4983 ns**
L3.2	108.0	12.5	139.5	13.6	105.5	99.5	8.3	40.0	3.8	50.5	5.4	0.90	0.07	40.0	0.0	10.5	79.2	28.7	0.0	6.88E-06
L3.3	444.0	53.9	542.0	58.5	510.5	510.5	52.4	3.0	0.2	9.0	0.7	0.90	0.06	31.5	28.5	6.0	33.3	5.8	5.3	8.58E-06
L3.4	288.5	35.5	264.0	26.2	295.0	261.5	24.8	2.5	0.2	9.0	0.9	0.86	0.02	2.5	0.0	6.5	27.8	0.9	0.0	**0.0274 ns**
L3.5	139.5	19.8	175.5	23.2	149.5	149.5	19.3	5.0	0.7	14.0	2.0	0.83	0.08	26.0	21.0	9.0	35.7	14.8	12.0	2.10E-05
L4.1	357.5	55.0	344.0	51.7	321.5	320.5	42.3	23.5	3.0	32.0	4.5	0.82	0.09	23.5	0.0	8.5	73.4	6.8	0.0	**0.4646 ns**
L4.2	281.0	49.5	323.0	49.8	298.5	298.5	46.1	4.0	0.6	15.0	2.4	0.78	0.06	24.5	20.5	11.0	26.7	7.6	6.3	**0.0824 ns**
L4.3	185.5	21.0	205.0	20.1	186.0	186.0	16.5	0.0	0.0	7.0	0.8	0.92	0.11	19.0	19.0	7.0	0.0	9.3	9.3	**0.0071 ns**
L5.1	232.0	21.7	209.5	19.0	217.5	203.5	19.4	6.0	0.7	18.5	3.8	0.97	0.13	6.0	0.0	12.5	32.4	2.9	0.0	**0.0436 ns**
L5.2	263.0	13.1	394.5	15.8	353.5	353.5	19.8	3.5	0.3	15.5	1.4	0.95	0.03	41.0	37.5	12.0	22.6	10.4	9.5	4.61E-24
L5.3	230.0	30.9	519.5	67.4	449.5	449.5	50.8	5.0	0.5	15.5	1.9	0.85	0.07	70.0	65.0	10.5	32.3	13.5	12.5	1.41E-14
L6.1	129.0	10.8	109.0	5.6	128.5	107.0	11.3	2.0	0.2	10.0	1.1	1.03	0.07	2.0	0.0	8.0	20.0	1.8	0.0	2.36E-07
L6.2	134.0	12.3	143.5	11.6	134.0	134.0	12.3	0.5	0.0	7.0	0.5	0.96	0.09	9.5	9.0	6.5	7.1	6.6	6.3	**0.0236 ns**
L6.3	169.0	18.9	194.0	29.1	200.5	189.0	25.7	5.0	0.6	13.0	1.6	1.32	0.17	5.0	0.0	8.0	38.5	2.6	0.0	**0.0111 ns**
L6.4	198.5	22.1	190.5	20.5	209.0	189.0	18.0	1.5	0.2	8.5	1.0	1.04	0.12	1.5	0.0	7.0	17.6	0.8	0.0	**0.2673 ns**
L6.5	166.5	22.0	191.5	25.7	172.5	172.5	27.3	1.0	0.2	10.0	1.4	0.78	0.06	19.0	18.0	9.0	10.0	9.9	9.4	**0.0080 ns**
L6.6	234.0	29.3	386.0	48.8	306.5	306.5	41.5	3.5	0.5	14.5	2.3	0.84	0.07	79.5	76.0	11.0	24.1	20.6	19.7	6.17E-12
L6.7	137.0	14.6	154.5	17.8	145.5	145.5	16.2	0.0	0.0	7.5	1.1	0.86	0.08	9.0	9.0	7.5	0.0	5.8	5.8	**0.0034 ns**
L6.8	154.0	17.3	182.5	19.3	171.5	171.5	24.7	7.5	0.8	13.0	1.4	0.89	0.09	11.0	3.5	5.5	57.7	6.0	1.9	4.49E-05
L6.9	97.0	13.8	135.0	18.5	152.5	134.5	19.5	0.5	0.1	8.5	1.2	1.29	0.18	0.5	0.0	8.0	5.9	0.4	0.0	5.58E-06
L6.10	204.0	18.9	233.0	32.8	218.5	218.5	19.4	2.0	0.2	6.5	0.7	0.96	0.12	14.5	12.5	4.5	30.8	6.2	5.4	**0.0129 ns**
L6.11	234.0	17.5	250.5	21.7	249.0	249.0	19.7	0.0	0.0	6.5	0.5	0.89	0.03	1.5	1.5	6.5	0.0	0.6	0.6	**0.0165 ns**
L6.12	128.0	14.7	130.5	15.8	122.0	122.0	16.6	0.5	0.1	7.0	0.9	0.82	0.06	8.5	8.0	6.5	7.1	6.5	6.1	**0.6312 ns**
P1.1	172.5	18.9	239.0	23.9	215.0	215.0	26.2	3.0	0.3	15.5	1.6	0.89	0.10	24.0	21.0	12.5	19.4	10.0	8.8	9.86E-08
P2.1	244.5	22.6	394.5	36.8	346.5	346.5	38.7	1.5	0.1	13.0	1.2	0.84	0.02	48.0	46.5	11.5	11.5	12.2	11.8	8.74E-15
P2.2	216.5	23.6	347.0	36.1	325.0	325.0	31.3	2.0	0.2	11.5	1.1	0.83	0.02	22.0	20.0	9.5	17.4	6.3	5.8	1.50E-13
P3.1	310.0	31.7	246.0	26.9	225.5	225.5	25.1	2.0	0.3	8.0	0.9	1.31	0.14	20.5	18.5	6.0	25.0	8.3	7.5	2.29E-06
P3.2	209.0	18.8	256.5	20.6	271.5	254.0	23.0	2.5	0.2	10.0	0.7	0.90	0.06	2.5	0.0	7.5	25.0	1.0	0.0	2.59E-08
P4.1	222.0	12.8	325.5	24.1	293.0	293.0	19.6	2.0	0.2	12.0	1.2	0.97	0.07	32.5	30.5	10.0	16.7	10.0	9.4	1.30E-12
P4.2	331.0	39.0	264.0	28.1	294.5	262.5	30.7	1.5	0.2	14.5	1.8	1.25	0.08	1.5	0.0	13.0	10.3	0.6	0.0	1.51E-06
P5.1	351.5	35.7	478.0	52.6	417.0	417.0	46.9	2.5	0.3	11.0	1.0	0.90	0.09	61.0	58.5	8.5	22.7	12.8	12.2	5.12E-08
P5.2	292.0	37.1	317.5	32.7	274.0	274.0	32.4	6.5	0.7	18.0	2.3	0.85	0.05	43.5	37.0	11.5	36.1	13.7	11.7	**0.0358 ns**
P5.3	217.0	18.1	276.5	29.7	212.0	212.0	20.5	1.5	0.1	10.0	1.0	0.87	0.05	64.5	63.0	8.5	15.0	23.3	22.8	1.05E-05
P5.4	230.5	44.8	259.5	49.0	240.0	240.0	45.8	1.5	0.3	8.0	1.3	0.74	0.07	19.5	18.0	6.5	18.8	7.5	6.9	**0.1022 ns**
P5.5	246.5	21.0	251.0	18.6	266.0	250.0	22.9	1.0	0.1	10.0	0.8	0.94	0.06	1.0	0.0	9.0	10.0	0.4	0.0	**0.4979 ns**
P5.6	185.0	28.3	190.0	30.3	174.5	174.5	26.7	2.5	0.4	15.0	2.5	0.81	0.09	15.5	13.0	12.5	16.7	8.2	6.8	**0.6291 ns**
P6.1	136.5	20.6	151.5	23.0	133.0	133.0	22.4	4.0	0.5	12.5	2.0	0.82	0.10	18.5	14.5	8.5	32.0	12.2	9.6	**0.0471 ns**
P6.2	239.5	20.1	191.0	19.6	180.0	180.0	16.9	1.0	0.1	13.5	1.5	0.89	0.06	11.0	10.0	12.5	7.4	5.8	5.2	2.96E-07
P6.3	143.0	12.6	127.5	17.2	122.5	122.5	14.0	3.0	0.3	11.5	1.1	0.92	0.10	5.0	2.0	8.5	26.1	3.9	1.6	**0.0043 ns**
P6.4	179.5	26.1	242.5	36.0	218.0	218.0	35.6	3.0	0.3	10.5	1.4	0.86	0.12	24.5	21.5	7.5	28.6	10.1	8.9	1.14E-06
P6.5	131.5	20.8	99.0	16.3	102.5	98.5	16.3	0.5	0.1	8.0	1.2	0.75	0.05	0.5	0.0	7.5	6.3	0.5	0.0	5.23E-05
P6.6	94.0	15.4	88.0	11.5	194.0	87.0	26.4	1.0	0.1	12.5	1.8	0.82	0.07	1.0	0.0	11.5	8.0	1.1	0.0	**0.2220 ns**
P6.7	195.0	28.4	219.5	34.8	207.5	207.5	31.5	0.5	0.1	9.5	1.5	0.78	0.08	12.0	11.5	9.0	5.3	5.5	5.2	**0.0283 ns**
P6.8	269.0	27.3	185.0	17.5	185.0	184.5	18.9	0.5	0.1	11.5	1.0	0.65	0.07	0.5	0.0	11.0	4.3	0.3	0.0	6.55E-12
P6.9	165.5	17.6	108.5	13.0	280.0	107.5	29.5	1.0	0.1	15.5	1.7	1.03	0.15	1.0	0.0	14.5	6.5	0.9	0.0	1.59E-08
P7.1	136.5	12.0	146.0	11.1	136.5	136.5	10.9	0.0	0.0	7.5	0.5	0.92	0.08	9.5	9.5	7.5	0.0	6.5	6.5	**0.0459 ns**
P7.2	341.5	25.3	458.5	36.6	420.5	420.5	25.6	1.5	0.1	6.5	0.4	1.03	0.09	38.0	36.5	5.0	23.1	8.3	8.0	2.20E-09
P7.3	489.5	48.5	405.5	38.9	417.0	404.0	39.1	1.5	0.1	5.5	0.5	0.88	0.06	1.5	0.0	4.0	27.3	0.4	0.0	2.29E-06
P7.4	216.0	36.0	308.5	39.9	266.0	266.0	37.6	4.0	0.5	12.0	1.5	0.86	0.12	42.5	38.5	8.0	33.3	13.8	12.5	1.76E-06
P8.1	128.0	6.5	155.5	9.3	165.5	152.5	9.4	3.0	0.2	15.0	0.8	0.91	0.02	3.0	0.0	12.0	20.0	1.9	0.0	1.64E-11
P8.2	194.0	9.4	397.5	40.8	282.0	282.0	15.3	2.0	0.1	20.0	1.0	1.01	0.07	115.5	113.5	18.0	10.0	29.1	28.6	2.19E-14
P9.1	201.5	18.4	287.0	26.9	260.5	260.5	23.7	1.5	0.1	14.0	1.3	0.88	0.06	26.5	25.0	12.5	10.7	9.2	8.7	3.67E-12
P9.2	181.5	10.7	183.0	15.5	236.5	182.0	13.7	1.0	0.1	13.0	0.7	0.94	0.06	1.0	0.0	12.0	7.7	0.5	0.0	**0.6778 ns**
P10.1	173.5	27.1	177.5	26.4	161.5	161.5	27.5	0.0	0.0	5.5	0.9	0.78	0.06	16.0	16.0	5.5	0.0	9.0	9.0	**0.6820 ns**

Isolate Code	Calculate Directly from Ct values													Estimated by subtraction			Percent			p-value differences 18S vs 28S [2]
	**18S**	**18S SD**[1]	**Total 28S**	**Total 28S SD**	**Uninserted 28S** (unadjusted)	**Uninserted 28S** (adjusted)	**Uninserted 28S SD**	**r*Pokey***	**r*Pokey* SD**	**Total *Pokey***	**Total *Pokey* SD**	**Tif to Gtp**	**Tif to Gtp SD**	**Inserted 28S** (total 28S - uninserted 28S)	**r*Inserts*** (total 28S - uninserted 28S - rPok)	**g*Pokey*** (total Pok - rPok)	**% rPok** (rPok/total Pok *100)	**% inserted 28S** (inserted 28S/total 28S *100)	**% r*Inserts*** (other inserted 28S/total 28S *100)	
P10.2	137.0	12.2	170.0	19.4	150.5	150.5	17.3	3.5	0.4	10.5	1.0	0.88	0.06	19.5	16.0	7.0	33.3	11.5	9.4	4.21E-06
P11.1	166.5	9.0	133.5	7.2	182.0	133.0	10.9	0.5	0.0	10.5	0.6	0.93	0.05	0.5	0.0	10.0	4.8	0.4	0.0	1.00E-13
P11.2	216.5	26.2	335.0	40.7	277.5	277.5	32.0	3.0	0.3	11.5	1.3	0.85	0.07	57.5	54.5	8.5	26.1	17.2	16.3	2.74E-11
P12.1	159.5	14.8	196.0	18.9	187.0	187.0	18.4	2.0	0.2	14.0	1.8	0.85	0.03	9.0	7.0	12.0	14.3	4.6	3.6	3.28E-06
P12.2	271.5	18.4	414.0	32.7	393.0	393.0	29.9	2.5	0.2	14.5	1.2	0.91	0.06	21.0	18.5	12.0	17.2	5.1	4.5	2.70E-15
P13.1	240.5	17.6	247.0	18.5	231.5	231.5	17.3	3.5	0.3	18.0	1.3	0.87	0.02	15.5	12.0	14.5	19.4	6.3	4.9	**0.2953 ns**
P13.2	122.5	10.7	163.0	15.2	152.5	152.5	14.9	2.5	0.2	11.5	1.0	0.90	0.07	10.5	8.0	9.0	21.7	6.4	4.9	2.26E-10
P14.1	448.0	46.6	724.5	56.4	637.0	637.0	53.2	0.5	0.1	10.5	1.0	1.00	0.11	87.5	87.0	10.0	4.8	12.1	12.0	4.48E-17
P14.2	385.5	21.8	495.5	29.3	428.5	428.5	18.6	0.0	0.0	8.0	0.5	0.94	0.03	67.0	67.0	8.0	0.0	13.5	13.5	5.77E-14
P15.1	124.0	13.0	133.5	12.9	135.0	131.5	14.7	2.0	0.2	10.0	1.0	1.08	0.13	2.0	0.0	8.0	20.0	1.5	0.0	**0.0316 ns**
P15.2	207.0	19.9	134.5	12.3	160.0	133.5	14.1	1.0	0.1	6.5	0.6	0.87	0.06	1.0	0.0	5.5	15.4	0.7	0.0	1.89E-11
P16.1	182.5	16.2	255.5	21.5	232.0	232.0	17.9	12.0	1.1	19.0	1.5	0.91	0.07	23.5	11.5	7.0	63.2	9.2	4.5	7.42E-13
log50-11	301.0	33.7	540.5	93.8	445.0	445.0	44.9	2.5	0.3	39.5	4.6	0.84	0.05	95.5	93.0	37.0	6.3	17.7	17.2	1.21E-09
log50-12	245.5	29.5	362.5	49.9	349.0	349.0	57.9	4.0	1.4	29.5	4.6	0.88	0.12	13.5	9.5	25.5	13.6	3.7	2.6	2.90E-09
Average Dpc	218.6		274.1		258.9	250.9		7.3		17.3		0.92		23.2	15.9	10.0	29.0	8.7	4.7	
Average Dpc (-3)	221.6		284.3		271.2	263.0		3.0		12.4		0.93		21.3	18.3	9.4	22.5	6.5	5.3	
Average Dpx	222.3		253.3		245.4	231.1		2.0		11.8		0.90		22.3	20.3	9.9	16.7	7.5	6.6	

1. SD = standard deviation
2. Values that are **NOT** significant after sequential Bonferroni correction are indicated with an **ns**

Uninserted 28S is adjusted when [uninserted 28S] or [uninserted 28S + rPokey] > total 28S
