# Supplementary material for: Copy number variation of ribosomal DNA and Pokey transposons in natural populations of Daphnia
Source: Mob DNA. 2012 Mar 5;3:4. doi: 10.1186/1759-8753-3-4 (PMC3315735; doi:10.1186/1759-8753-3-4)

Additional File 3 – *Tif* and *Gtp* Cloning Experiment

Objective

Tif:Gtp ratios in the 69 *Daphnia* isolates vary from 0.65 to 1.32 with a mean of 0.9 in *D. pulex* and 0.92 in *D. pulicara* (Figure 1). The expected value is 1 so this is somewhat low. However, the distribution is normal with the exception of five outliers. There could be variation in the genes that affects primer annealing during qPCR, but it is also possible that one of the two reference genes may be present in three copies instead of the two expected, in which case we would expect a ratio of 0.67 (3 *Gtp*) or 1.5 (3 *Tif*). To test these possibilities, we PCR amplified, cloned and sequenced the *Tif* and *Gtp* genes from four *Daphnia* isolates: one with a low TG ratio (0.70), one with a high TG ratio (1.31) and two with ratios near the mean (0.81, 0.89).

Cloning

A 560 bp region of the Tif and Gtp genes from the genomic DNA of isolate L1.1, P1.1, P3.1, and P5.6 were PCR amplified with primers G6 F [5'- AGAAATTCAACATGCCCAAGA] and G12 R [5'- CGTCGACGAAGTTGACAGTATC] and F6 F [5'- CGTTTCGAATTGGCTTACTGA] and F12 R [5'- CATGGTTATCTGTCTACGTCTTGAA], respectively [1]. A 25 µL PCR reaction was run for all four isolates for both *Tif* and *Gtp*. The 25 µL contained 1X Phusion High-Fidelity Reaction Buffer (New England Biolabs), 1.25 pmol of each primer, 0.08 mM dNTPs, 0.2 units of Phusion High-Fidelity *Taq* DNA polymerase (New England Biolabs), and approximately 40 to 60 ng of DNA.

All reactions were run on a T100 Thermo Cycler (BioRad). The PCR protocol for *Tif* was 2 min at 94ºC, 25 cycles of 94ºC for 30 sec, 55ºC for 30 sec and 72ºC for 2 min. This PCR product (2uL) was used as template for a secondary PCR under the same conditions. In attempt to reduce the number of PCR recombinants, the following PCR reaction was used for *Gtp*: 2 min at 94ºC, 25 cycles of 94ºC for 30 sec, 55ºC for 30 sec and 72ºC for 90 sec, and 10 cycles of 94ºC for 30 sec, 55ºC for 30 sec and 72ºC for 90 sec plus an additional 20 sec/cycle, with a final elongation at 72ºC for 5 min.

PCR products were verified by running them on a 1% TAE agarose gel, stained with GelRed™ Nucleic Acid Gel Stain (Biotium) and visualized under UV light. The PCR product was cloned into pSC-B-amp/kan using the StrataClone Blunt PCR Cloning Kit (Agilent Technologies) according to manufacturer's instructions.

Sequencing

Colonies were screened by PCR with M13F [5'-GTTGTAAAACGACGGCCAGTG] and M13R [5'-CAGGAAACAGCTATGACCATG]. Colonies were added to 10 µL of PCR grade H_2O, heated at 99.9°C for 3 min and 1 µL was used for PCR. The 25 µL PCR reaction contained 1X buffer (100mM Tris-HCl, pH 8.3; 200mM KCl), 1.5mM $MgCl_2$, 0.04mM dNTPs, 1pmol of each primer, 0.1 units GenScript Taq (GenScript). Reactions were run on a T100 Thermo Cycler (BioRad) for 2 min at 94ºC, 35 cycles of 94ºC for 30 sec, 55ºC for 30 sec and 72ºC for 1 min with a final elongation at 72ºC for 5 min. PCR products were verified by running them on a 1% TAE agarose gel, stained with GelRed™ Nucleic Acid Gel Stain (Biotium) and visualized under UV light.

The PCR product of twelve clones for each gene from each isolate was sequenced. The 12 µL sequencing reactions contained 0.3 µL BigDye Terminator Mix v3.1 (Applied Biosystems), 1 µL sequencing buffer (Applied Biosystems), 10 pmol of M13F primer, and 2 µL PCR product. Reactions were run on a PTC-100 Thermocycler (MJ Research, Waltham, MA) for 1 min at 96°C followed by 30 cycles of 96°C for 20 sec, 55°C for 20 sec, and 60°C for 4 min. Reactions were sent to the Genomics Facility at the University of Guelph and resolved on an ABI 3730 DNA Analyzer (Applied Biosystems).

Analysis

Sequences were edited in CLC Workbench (CLC bio). MEGA 5.0 was used to align the sequences and construct Neighbor-joining trees using the Maximum Composite Likelihood method to estimate pairwise sequence divergence [2]. Sequences that were recombinants of two alleles due to template switching during amplification, and sequences with unique single nucleotide changes within an isolate (singletons) were removed from the analysis.

Results

Neighbor-joining trees of up to 12 clones from each isolate show that isolate P3.1 has 3 *Tif* alleles (Figure 2). Although the Tif:Gtp ratio of isolate L1.1 is only 0.70, there is no evidence that it contains 3 *Gtp* alleles (Figure 3). Based on the frequency distribution of Tif:Gtp ratios (Figure 1), we conclude that the isolate with a ratio of 0.65 has 3 *Gtp* alleles. Gene number estimates in isolates with 3 *Tif* or 3 *Gtp* genes were corrected by multiplying them by 1.5.

Examination of the gene sequences shows that the qPCR primer sites are highly conserved. There are no more than 2 substitutions in the primer sites of the 8 *Gtp* and 9 *Tif* alleles sequenced, and they are always transitions that occur at least 16 nt from the 3' end of the primer, and are thus unlikely to have a substantial effect on priming efficiency.

References

1. Omilian AR, Lynch M: **Patterns of intraspecific DNA variation in the *Daphnia* nuclear genome.** *Genetics* 2009, **183**:325-326.

2. Tamura K, Peterson D, Peterson N, Stecher G, Nei M, Kumar S: **MEGA5: Molecular Evolutionary Genetics Analysis using Maximum Likelihood, Evolutionary Distance, and Maximum Parsimony Methods.** *Mol Biol Evol* 2011, **28**:2731-2739.

Figure 1. Histogram of *Tif:Gtp* ratios in 69 isolates of *Daphnia*. The mean is 0.91. There are 4 isolates with 3 *Tif* to 2 *Gtp* genes (3:2) and 1 isolate with 2 *Tif* to 3 *Gtp* genes (2:3).

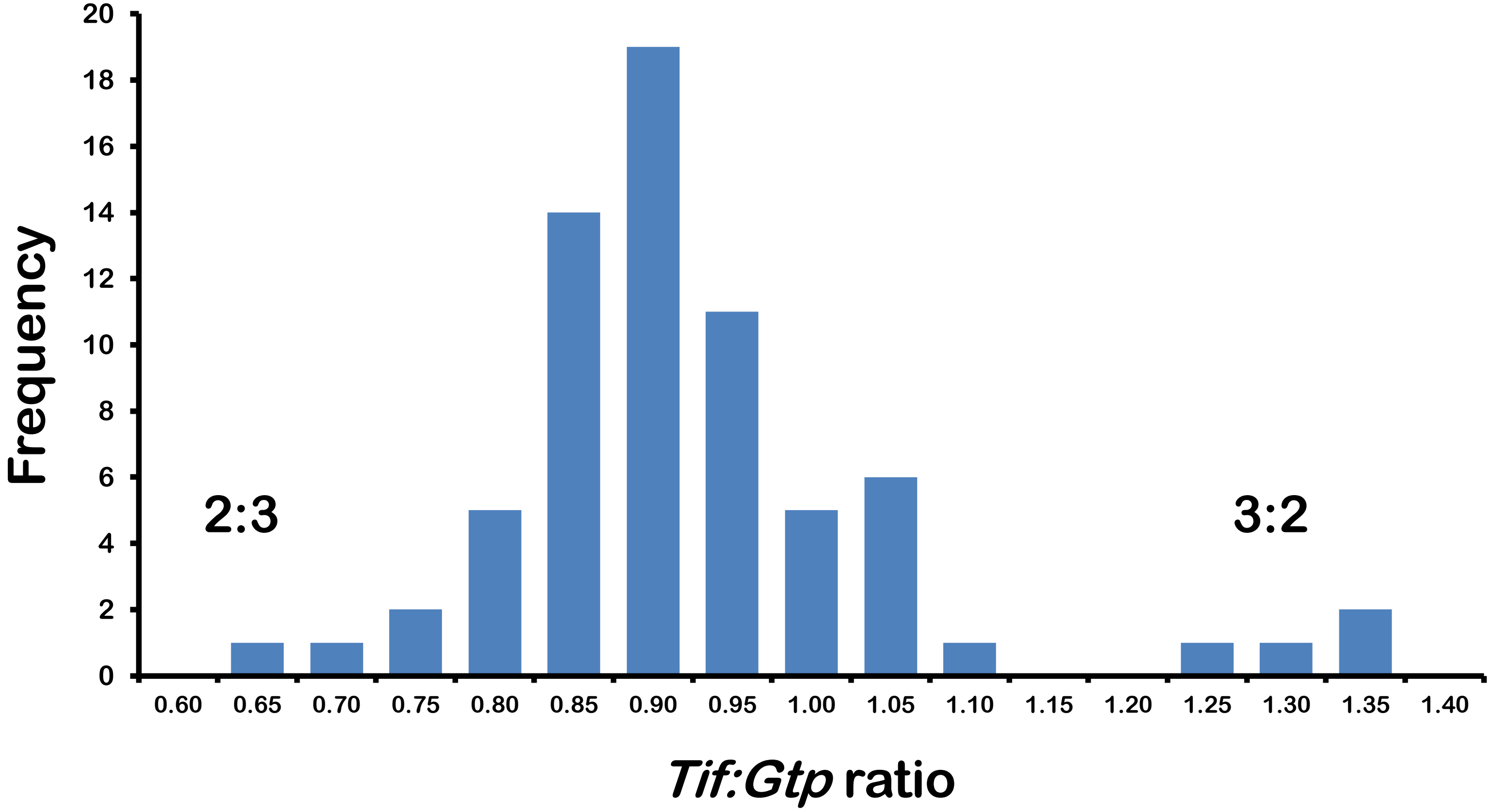

Figure 2. Neighbor-joining trees of *Tif* plasmid clones from 4 *Daphnia* isolates, L1.1, P5.6, P1.1 and P3.1. T:G = *Tif:Gtp* ratio as estimated using qPCR.

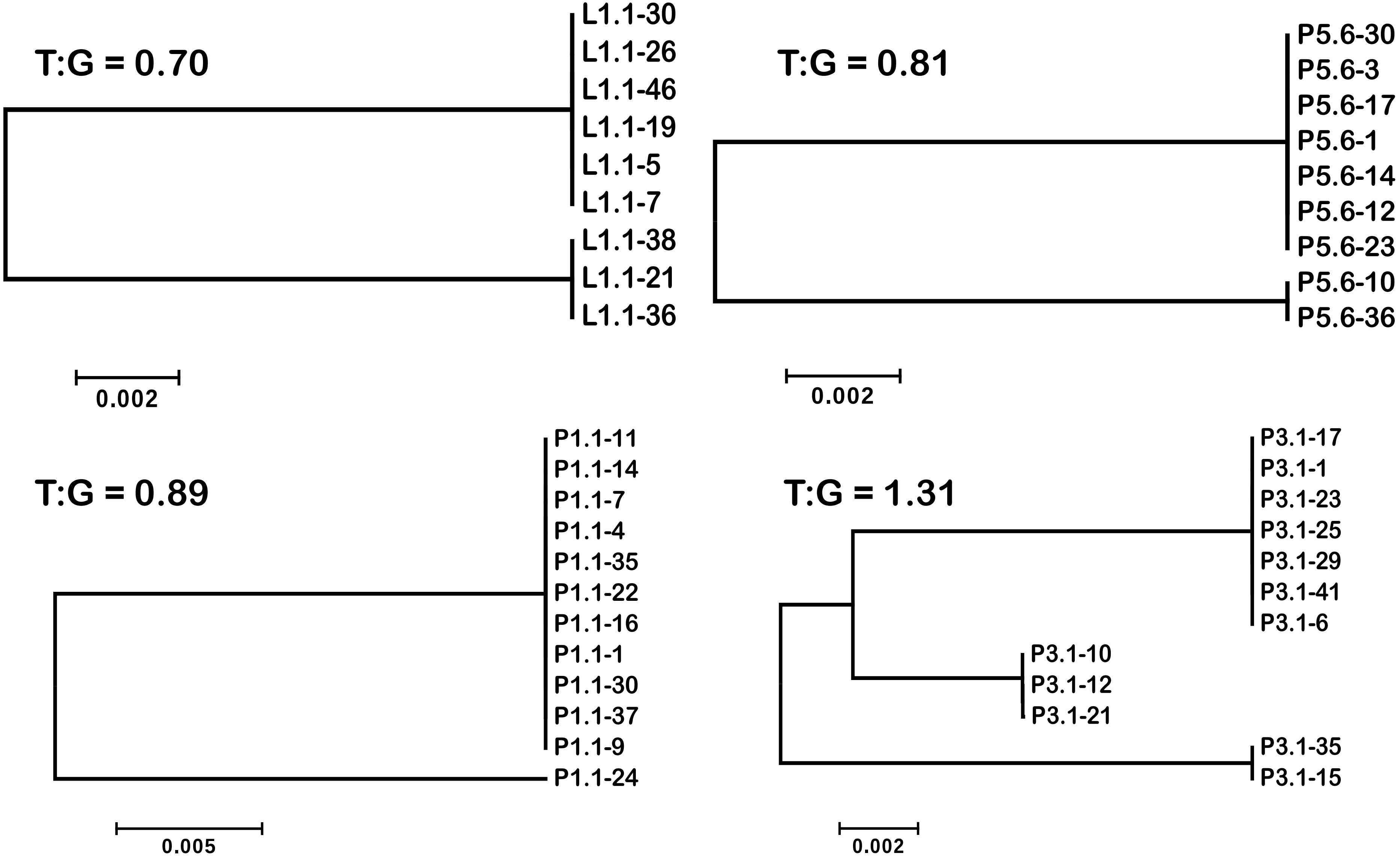

Figure 3. Neighbor-joining trees of *Gtp* plasmid clones from 4 *Daphnia* isolates, L1.1, P5.6, P1.1 and P3.1. T:G = *Tif:Gtp* ratio as estimated using qPCR.

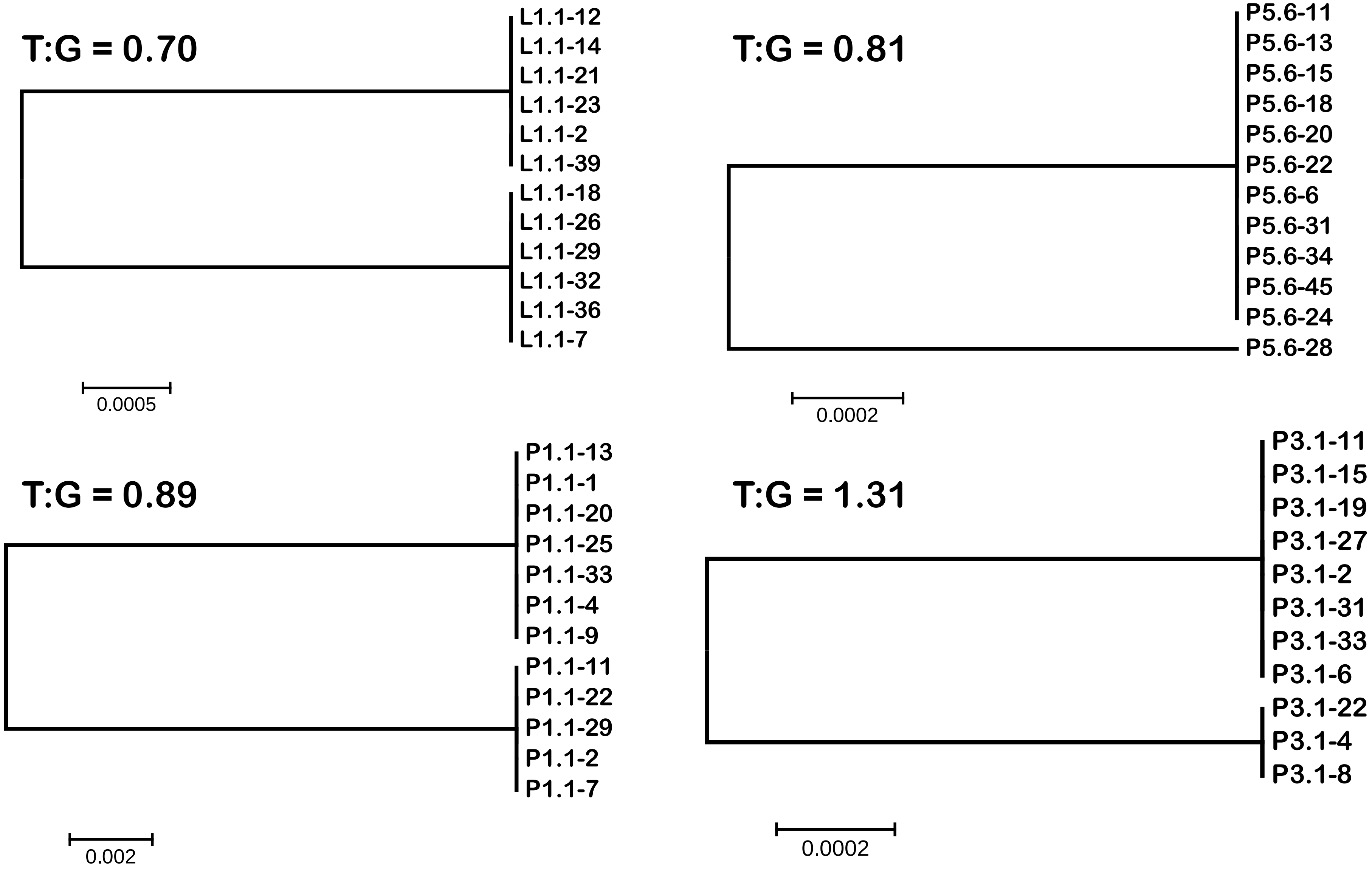

Supplement: Additional file 3 — Tif and Gtp cloning experiment. This PDF file describes the cloning and sequencing of Tif and Gtp genes from four Daphnia isolates with a range of Tif:Gtp ratios. The purpose of this work was to determine if isolates with very low or very high Tif:Gtp ratios possess three alleles at one of the loci. [file 1759-8753-3-4-S3.PDF]
