# Supplementary material for: Copy number variation of ribosomal DNA and Pokey transposons in natural populations of Daphnia
Source: Mob DNA. 2012 Mar 5;3:4. doi: 10.1186/1759-8753-3-4 (PMC3315735; doi:10.1186/1759-8753-3-4)
Supplement: Additional file 4 — Histograms of haploid rRNA gene and insertion number. This is a PDF file. (a) Haploid 18S and 28S gene number in each Daphnia isolate. Vertical lines are standard errors. Differences that are not significant after sequential Bonferroni correction are indicated by "ns". (b) Haploid number of rPokey and 28S with and without inserts in each Daphnia isolate. u28S are uninserted 28S genes, rPokey are inserted in 28S, rInserts are inserts other than rPokey in 28S. The number of rInserts was calculated as (total 28S-uninserted 28S-rPokey). [file 1759-8753-3-4-S4.PDF]

Additional File 4.
Haploid rRNA gene and insertion number.

[a] Haploid 18S and 28S gene number in each *Daphnia* isolate. Vertical lines are standard errors. Differences that are NOT significant after sequential Bonferroni correction are indicated by “ns”.

[b] Haploid number of r*Pokey* and 28S genes with and without insertions in each *Daphnia* isolate.
u28S are uninserted 28S genes
r*Pokey* are inserted in 28S genes.
r*Inserts* are insertions other than r*Pokey* in 28S genes.
r*Inserts* were calculated as [total 28S – uninserted 28S – r*Pokey*].

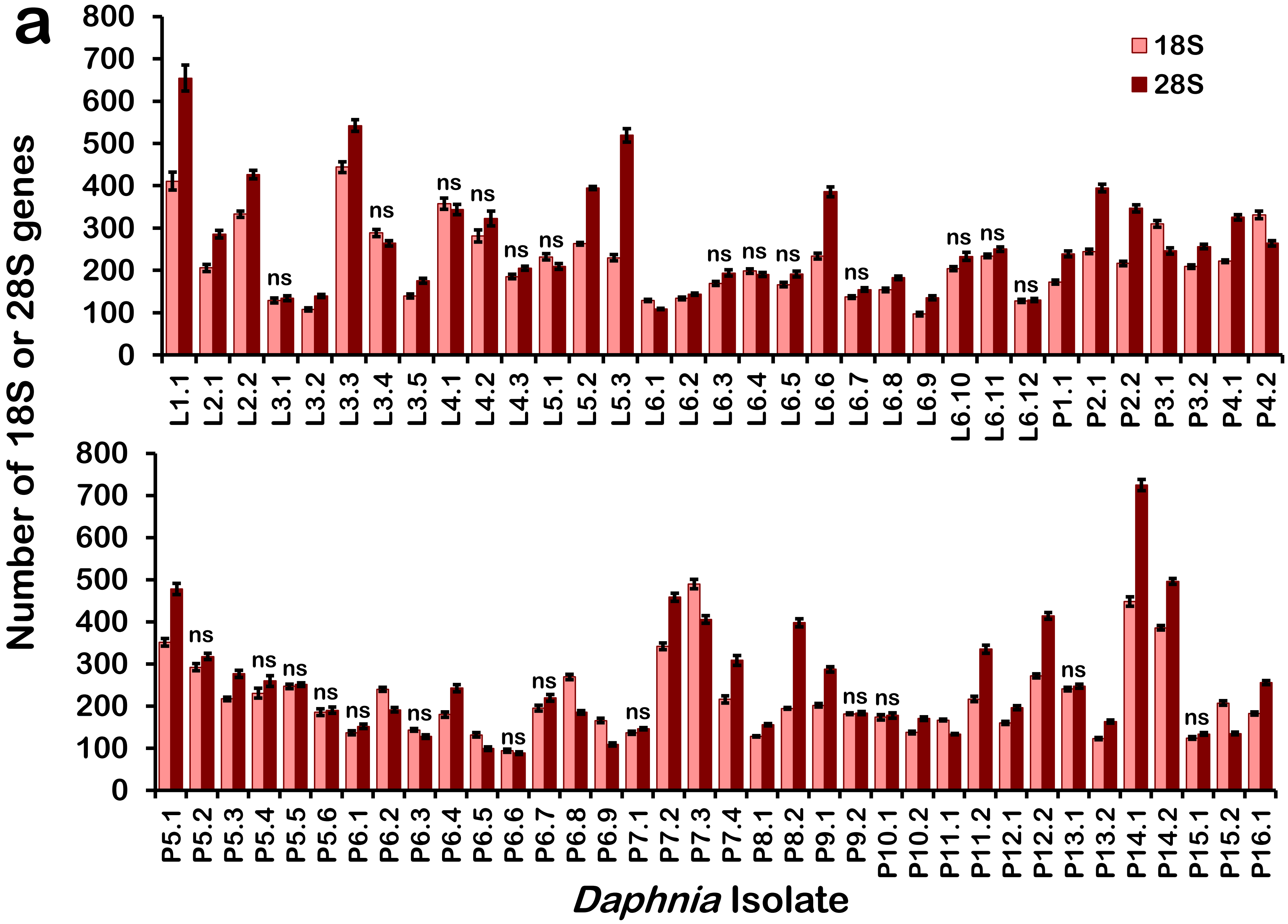

a
Number of 18S or 28S genes
18S
28S
ns
L1.1 L2.1 L2.2 L3.1 L3.2 L3.3 L3.4 L3.5 L4.1 L4.2 L4.3 L5.1 L5.2 L5.3 L6.1 L6.2 L6.3 L6.4 L6.5 L6.6 L6.7 L6.8 L6.9 L6.10 L6.11 L6.12 P1.1 P2.1 P2.2 P3.1 P3.2 P4.1 P4.2
P5.1 P5.2 P5.3 P5.4 P5.5 P5.6 P6.1 P6.2 P6.3 P6.4 P6.5 P6.6 P6.7 P6.8 P6.9 P7.1 P7.2 P7.3 P7.4 P8.1 P8.2 P9.1 P9.2 P10.1 P10.2 P11.1 P11.2 P12.1 P12.2 P13.1 P13.2 P14.1 P14.2 P15.1 P15.2 P16.1
Daphnia Isolate

b

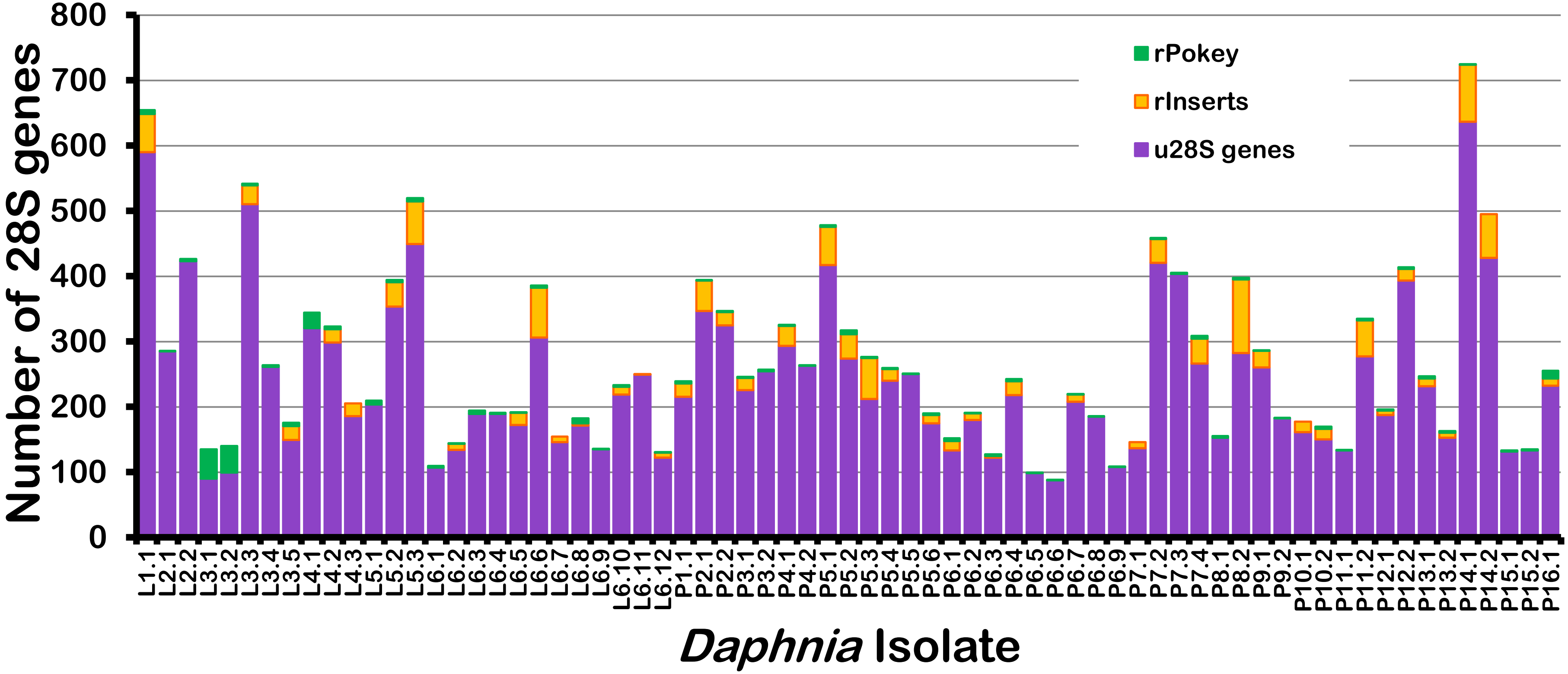

rPokey
rInserts
u28S genes
Number of 28S genes
0
100
200
300
400
500
600
700
800
L1.1
L2.1
L2.2
L3.1
L3.2
L3.3
L3.4
L3.5
L4.1
L4.2
L4.3
L5.1
L5.2
L5.3
L6.1
L6.2
L6.3
L6.4
L6.5
L6.6
L6.7
L6.8
L6.9
L6.10
L6.11
L6.12
P1.1
P2.1
P2.2
P3.1
P3.2
P4.1
P4.2
P5.1
P5.2
P5.3
P5.4
P5.5
P5.6
P6.1
P6.2
P6.3
P6.4
P6.5
P6.6
P6.7
P6.8
P6.9
P7.1
P7.2
P7.3
P7.4
P8.1
P8.2
P9.1
P9.2
P10.1
P10.2
P11.1
P11.2
P12.1
P12.2
P13.1
P13.2
P14.1
P14.2
P15.1
P15.2
P16.1
Daphnia Isolate
