# Supplementary material for: Copy number variation of ribosomal DNA and Pokey transposons in natural populations of Daphnia
Source: Mob DNA. 2012 Mar 5;3:4. doi: 10.1186/1759-8753-3-4 (PMC3315735; doi:10.1186/1759-8753-3-4)
Supplement: Additional file 5 — qPCR primers. This PDF file provides sequences for qPCR primers, as well as the threshold value and the percent amplification efficiency (PAE) for each primer pair. [file 1759-8753-3-4-S5.PDF]

Additional File 5. qPCR primers

Gene	Primer	Primer sequence	Amplicon Size	Threshold[1]	Slope	Percent Amplification Efficiency[2]
18S	18S1864F	5'-ccg cgt gac agt gag caa ta	50	0.2	-3.476	0.9556
	18S1913R	5'-ccc agg aca tct aag ggc atc				
total 28S	28S3054F	5'-ggt agc caa atg cct cgt ca	150	0.3175	-3.593	0.9246
	28S3204R	5'-gag tca agc tca aca ggg tct tct ttc cc				
uninserted 28S	28S2974F	5'-ctg ccc agt gct ctg aat gtc aaa gtg aag	131	0.3064	-3.682	0.9023
	28S3104R	5'-gtt aat cca ttc gtg cgc g				
r*Pokey*	Pok6456F	5'-gac aac ggt ggc cga aac gcg g	192	0.3339	-3.709	0.8957
	28S3104R	5'-gtt aat cca ttc gtg cgc g				
total *Pokey*	Pok6456F	5'-gac aac ggt ggc cga aac gcg g	122	0.3011	-3.636	0.9136
	Pok6578R	5'-gat ggt cgg att cga ttg aat gct cg				
Tif	TIF392F	5'-gac atc atc ctg gtt ggc ct	50	0.2	-3.499	0.9493
	TIF442R	5'-aac gtc agc ctt ggc atc tt				
Gtp	GTP385R	5'-tat tca gca tgg aga gac ggc	50	0.2	-3.546	0.9369
	GTP435R	5'-gat gtc gac tga cgc tgg aa				

1. Calculated as 0.2 x 2 ^[1-(50/length in bp)].

2. Calculated as ln [10^(-1/slope)] / ln (2)
