# Supplementary material for: Copy number variation of ribosomal DNA and Pokey transposons in natural populations of Daphnia
Source: Mob DNA. 2012 Mar 5;3:4. doi: 10.1186/1759-8753-3-4 (PMC3315735; doi:10.1186/1759-8753-3-4)
Supplement: Additional file 6 — Standard curve analysis for qPCR primers. This PDF file provides details of the standard curve experiments used to validate the qPCR primers, including a description of the plasmid clones used as templates. [file 1759-8753-3-4-S6.PDF]

Additional File 6 – Standard Curve Experiments

Standard curve experiments as described in [1] were conducted to determine the percent amplification efficiency (PAE) of each qPCR primer pair. PCR amplicons generated from plasmid DNA containing inserts of our regions of interest (Table 1) were used as DNA templates for this analysis.

Cloning the 28S gene

A 2.6 kb region of the 28S rRNA gene from *Daphnia pulex* genomic DNA was PCR amplified with primers 28S1506F [5'-ATCCGCTAAGGAGTGTGTAACAACTCACC] and 28S4102R [5'-CCGGACGTAGCCTCGCACCAC]. The 25 µL PCR reaction contained 1X Phusion High-Fidelity Reaction Buffer (New England Biolabs), an extra 0.5 mM of $MgCl_2$, 1.25 pmol of each primer, 0.08 mM dNTPs, 0.1 units of Phusion High-Fidelity *Taq* DNA polymerase (New England Biolabs), and approximately 5 ng of DNA. The reaction was run on a PTC-100 Thermocycler (MJ Research, Waltham, MA) for 2 min at 94ºC, 20 cycles of 94ºC for 30 sec, 55ºC for 30 sec and 72ºC for 3 min, and 10 cycles of 94ºC for 30 sec, 55ºC for 30 sec and 72ºC for 3 min 20 sec plus an additional 20 sec/cycle.

PCR amplicons were verified by running them on a 1% TAE agarose gel, staining it with GelRed™ Nucleic Acid Gel Stain (Biotium) and visualizing it under UV light. The PCR product was cloned into pSC-B-amp/kan using the StrataClone Blunt PCR Cloning Kit (Agilent Technologies) according to manufacturer's instructions. Plasmid DNA was extracted for each of the five plasmids using the High Pure Plasmid Isolation Kit (Roche) according to manufacturer's instructions. DNA concentration was measured using a NanoDrop® ND-8000 spectrophotometer.

Generation of PCR amplicons

Plasmid DNA (1 to 30 ng) was used as template in PCR reactions. Five 25 µL PCR reactions were run for each template. The 25 µL PCR reactions contained 10X Buffer (100mM Tris-HCl, pH 8.3; 200mM KCl), 1.5 mM of $MgCl_2$, 1.25 pmol of each primer, 0.04 mM dNTPs, 0.1 units of GenScript *Taq* DNA polymerase (GenScript). The

reaction was run on a T100 Thermo Cycler (BioRad) for 2 min at 94ºC, 35 cycles of 94ºC for 30 sec, 55ºC for 30 sec and 72ºC for 90 sec, and a final extension at 72ºC for 5 min. Primers and product sizes are listed in Table 2. PCR amplicons were verified by running them on a 1% TAE agarose gel, staining it with GelRed™ Nucleic Acid Gel Stain (Biotium) and visualizing it under UV light. The PCR amplicons were cleaned up using the E.Z.N.A Micro-Spin DNA Clean-Up Kit (Omega Bio-Tek) as per manufacturer's instructions. DNA concentration was measured using a NanoDrop® ND-8000 spectrophotometer.

Standard Curve

Triplicate qPCR reactions containing 500000, 50000, 5000 and 500 copies per microliter of each PCR amplicon were used as the template for each qPCR primer pair. Each qPCR reaction had a final volume of 20 µL, and contained 1X Power SYBR® Green (PCR Master Mix, Applied Biosystems), 0.25 pmol of each primer and 5 µL of PCR product template DNA. Amplification was done on the StepOnePlus (Applied Biosystems) using the default qPCR program of 95ºC for 10 min, 40 cycles of 95ºC for 15 sec and 60ºC for 1 min. In addition, the temperature was increased from 60ºC to 95ºC in 0.3ºC increments to create a dissociation curve.

The baseline was set by the StepOne v2.1 software (Applied Biosystems) but the threshold was set depending on amplicon size. The threshold of the smallest amplicons (50 nt) was set to 0.2. The threshold of the other amplicons was calculated as 0.2 x 2^[1-(50/length in bp)]. We plotted mean C_T relative to the log of template copy number, and then calculated the slope of the best fit line. The slope was used to calculate the amplification efficiency (AE) as AE = 10^(-1/slope) [2]. AE was used to calculate the percent amplification efficiency (PAE) as PAE = ln(AE)/ln(2) [2]. The PAE was used to calculate the copy number and correct for amplification efficiency differences between primer pairs. Slope and PAE values for each primer pair are reported in Additional File 5.

References

1. Applied Biosystems: **User bulletin no. 2, ABI 7700 SDS. Relative Quantitation of Gene Expression.** October 2001.

2. Yuan JS, Wang D, Stewart CN: **Statistical methods for efficiency adjusted real-time PCR quantification.** *Biotechnol J* 2008, **3**:112-123.

3. Crease TJ, Colbourne JK: **The unusually long small-subunit ribosomal RNA of the crustacean, *Daphnia pulex:* sequence and predicted secondary structure.** *J Mol Evol* 1999, **46**:307-313.

4. Penton EH, Crease TJ: **Evolution of the transposable element *Pokey* in the ribosomal DNA of species in the subgenus *Daphnia* (Crustacea: Cladocera).** *Mol Biol Evol* 2004, **21**:1727-1739.

5. Omilian AR, Lynch M: **Patterns of intraspecific DNA variation in the *Daphnia* nuclear genome.** *Genetics* 2009, **183**:325-326.

Table 1. Plasmids used to generate templates for qPCR standard curve experiments.

Plasmid clone	Description	qPCR target	Plasmid vector	Plasmid insert size (bp)	Total plasmid size (bp)	Reference
18S	18S gene	➢ total 18S genes	pBluescript-KS+	3789	6750	3
r*Pokey*	*Pokey* 3' end and 28S gene downstream	➢ r*Pokey* elements<br>➢ total *Pokey* elements	pCR4-TOPO	1726	5682	4
28S	partial 28S gene	➢ total 28S genes<br>➢ uninserted 28S genes	pSC-B-amp/kan	2570	6842	this study
Tif	partial *Tif* gene	➢ *Tif* reference gene	pCR4-TOPO	561	4517	5
Gtp	partial *Gtp* gene	➢ *Gtp* reference gene	pCR4-TOPO	538	4494	5

Table 2. PCR primers used to generate templates for qPCR standard curve experiments.

Plasmid clone	Primer	Primer Sequence (5'-3')	PCR amplicon size (bp)
18S	18S 1522 F	att ccg ata acg aac gag	482
	18S 2004 R	tgg gga tca ttg cag tcc cca atc	
r*Pokey*	Pok 6172 F	tgg tcg atg gta aag acc tca acg tc	479
	28S 3104 R	gtt aat cca ttc gtg cgc g	
28S	28S 2292 F	acg cga cac aaa ccg gag aag	912
	28S 3204 R	gag tca agc tca aca ggg tct tct ttc cc	
Tif	M13 F	gtt gta aaa cga cgg cca gtg	760
	M13 R	cag gaa aca gct atg acc atg	
Gtp	M13 F	gtt gta aaa cga cgg cca gtg	760
	M13 R	cag gaa aca gct atg acc atg	
